# EEG-Based Engagement Monitoring in Cognitive Games

**DOI:** 10.3390/s25072072

**Published:** 2025-03-26

**Authors:** Yusuf Ahmed, Martin Ferguson-Pell, Kim Adams, Adriana Ríos Rincón

**Affiliations:** 1 Department of Occupational Therapy, Faculty of Rehabilitation Medicine, University of Alberta, 8205 114 St NW, Edmonton, AB T6G 2G4, Canada; 2 Department of Biomedical Engineering, Faculty of Engineering and Technology, University of Ilorin, Ilorin 1515, Nigeria; 3 Faculty of Rehabilitation Medicine, University of Alberta, 8205 114 St NW, Edmonton, AB T6G 2G4, Canada

**Keywords:** engagement, older adults, computerised cognitive games, EEG, machine learning (ML), cognitive decline, dementia

## Abstract

Cognitive decline and dementia prevention are global priorities, with cognitive rehabilitation games showing potential to delay their onset or progression. However, these games require sufficient user engagement to be effective. Assessing the engagement through questionnaires is challenging for the individuals suffering from cognitive decline due to age or dementia. This study aims to explore the relationship between game difficulty levels, three EEG engagement indices (*β*/(*θ* + *α*), *β*/*α*, 1/*α*), and the self-reported flow state scale score during video gameplay, and to develop an accurate machine learning algorithm for the classification of user states into high- and low-engagement. Twenty-seven participants (nine older adults) played a stunt plane video game while their EEG signals were recorded using EPOCX. They also completed the flow state scale for occupational tasks questionnaire after the easy, optimal, and hard levels of gameplay. Self-reported engagement scores significantly varied across the difficulty levels (*p* = 0.027), with the optimal level yielding the highest scores. Combining the three EEG indices achieved the best performance, with F1 scores of 89% (within-subject) and 81% (cross-subject). Engagement classification F1 scores were 90% for young adults and 85% for older adults. The findings provide preliminary data that supports using EEG data for engagement analysis in adults and older adults.

## 1. Introduction

Cognitive decline and dementia prevention and rehabilitation have been flagged by the World Health Organisation as global mental health priorities [1]. This report was based on the projection that the global population of older adults aged above 60 years old would double by 2050 [1]. Dementia contributes significantly to disability, and its debilitating effects impact the lives of the patient, their relatives, and the community. The complexity and burden of care tend to increase as the volume of the older population increases and the disease progresses [2]. Its attendant economic implications have been projected to double in 2040, while an estimated EUR 232 billion was reported to have been spent in Europe in 2015 [3]. Global expenses on dementia have been projected to reach USD 1.6 trillion by 2050 [4].

In Canada, according to a 2022 Landmark study [5], the population of people living with dementia will almost triple its 2020 value by 2050. By 2030, the annual incidence of dementia is projected to increase to 187,000/year, reaching a population of one million [5]. The more recent Landmark 2024 report of the Alzheimer’s Society of Canada expects the population of people living with dementia to rise by 187% in the next 30 years [6].

The Landmark study charted the course for dementia risk reduction, among which is a strategy to delay the onset of dementia. The report explained that a delay could significantly reduce dementia risk; a delay of one year could lead to 500,000 fewer cases by 2050, according to their simulation. The five- and ten-year delays are projected to result in 2,287,800 and 4,030,700 fewer cases, respectively. In the report, playing cognitive games was highlighted as one of the ways to achieve this delay, in addition to reading, writing, and physical exercise [5]. Even though this projection seems plausible, it has been corroborated by the longitudinal study by Wilson et al. [7], which concluded that a cognitively active life at an older age could delay the onset of dementia by at most five years. People living with dementia (PwD) often experience a cognitive decline, including long processing time, poor memory, and behavioural changes, such as impatience and irritability, which makes engagement challenging to achieve.

Engagement is a state of concentration, immersion, or absorption in a task cognitively, behaviourally, and emotionally [8,9,10]. Engagement is often associated with losing a sense of time awareness due to a high immersion and interest in the task at hand. Engagement can be positive when the person feels absorbed and wants to continue performing the activity (as the effort matches the task), but it can also be negative when the person feels bored due to the challenge being too small, or frustrated because the challenge is too much to match their skills which causes them to want to discontinue playing [11].

In addition, engagement in video games has been linked to the flow state, a concept obtained from the flow theory. The flow theory proposes that a flow state is achieved when someone is engrossed in a task to the extent that they lose track of time [12]. In the context of playing computer games, achieving this state makes the player want to play more. The flow theory was used as the theoretical framework, guiding the evaluation of engagement in this study as it provides a compelling high-level model that several researchers and game developers have used to examine engagement in gaming environments [10,13,14]. Flow is particularly well suited to describing the positive psychological state when the individuals engage in experiences perceived as challenging yet still obtainable, often leading to high engagement [15]. In addition, an adequate playing time of computerised cognitive games is positively associated with improved cognitive skills [16].

Quantifying engagement has been a significant area of research over the decades, being a complex and multidimensional construct. It is interesting to note that even though engagement is used extensively when describing a player’s behaviour during gameplay, the dimensions of engagement are quite complex, and it is difficult to determine whether a person is engaged [11]. According to the literature, there are no globally agreed attributes or theories for determining engagement, hence the existence of the various methodologies and approaches to engagement measurement [15,17].

The traditional gold standard for measuring engagement is answering a questionnaire that seeks to evaluate the individual’s feelings about the perceived level of engagement [15]. There are several questionnaires designed for this purpose, such as the game engagement questionnaire (GEQ) [18], the flow state scale of occupational therapy [19], and the user engagement scale (UES) [20], which have been validated, and other ones that are still in the development process [9,21]. However, this approach is subjective and retrospective (not real-time), which could create bias.

Ageing is often associated with a minor or major decline in cognitive abilities such as processing speed and working memory, among others. There has been an increased interest in ageing-in-place, which often requires home-based therapies, among which is the use of cognitive games. Studies have shown that cognitive games have the potential to maintain cognition and delay the onset of a cognitive decline among older adults [1,22,23]. Researchers have stressed that adequate user engagement is needed in these games for them to be an effective intervention [1,24]. It has been observed that assessing the engagement through questionnaires is difficult for those who are not able to understand the questionnaires or remember their play experience due to age or cognitive decline. Hence, alternative methods of assessing engagement are needed, such as using physiological markers. The common behavioural markers of game engagement, such as facial expression, eye blinks, and body movements, are found to be more effective in young adults and less effective in older adults [25]. Physiologically, there is a difference in these features of engagement for older and young adults. The absence of an older adult data set is an additional factor. Hence, a physiological signal, such as an electroencephalogram (EEG) in a passive neurofeedback brain–computer interface (BCI) paradigm, holds a strong potential for the assessment and sustainment of engagement in cognitive games.

With the growing number of older adults using computer games [26,27], a passive neurofeedback BCI paradigm could help sustain the engagement of older adults when playing these rehabilitative, cognitive games at home. The recent advancement in technology has led to an increase in the volume of commercial BCI systems on the market [1]. However, the first step towards a passive BCI adaptive game control is an adequate data acquisition system and an accurate machine learning (ML) algorithm for the engagement assessment. Thus, this study aims to answer the following research questions:

(1) Are there differences in the (self-reported) levels of engagement across the game levels?
(2) Are there any differences in the EEG engagement indices across the game difficulty levels?
(3) How accurately can the reported engagement be classified from the EEG data using the within-subject and cross-subject approaches?
  (3.1) Do the older adults’ and young adults’ EEG data compare with each other in terms of performance reports?
  (3.2) Which classifier is better in performance?

## 2. Related Works

Automatic measurement of engagement requires biomarkers, which serve as features that contain expressions indicative of engagement and can be used to quantify it. These markers of engagement can be found across the three dimensions of engagement, namely the cognitive, emotional, and behavioural dimensions. Physiological signals such as EEG, extracted from the scalp by EEG electrodes/sensors, electrooculography (EOG), extracted from eye movements, electrodermal signals, otherwise called skin conductance, extracted from the skin, and electrocardiography (ECG), extracted from the electrical activities of the heart, among others, have been used to explore the levels of engagement [28,29,30,31]. The EEG is one of the most explored schemes in classifying engagement during gameplay [32]. EEG’s popularity in engagement measurement is linked to its strong correlation with emotion and cognition, which are the dimensions of engagement expression, and to its high temporal resolution [33].

EEG and other automatic engagement analyses have employed ML extensively in the area of human–computer interactions for task classification, learning concentration classification, and workload and engagement classification [34,35,36,37,38,39,40]. The adoption of ML in these studies is due to its capacity to handle large, complex, and high-dimensional data, and to synthesise insights from them. The learning process is computational, which makes ML worthwhile in automatic systems.

Automatic engagement detection and measurement in computerised video games for rehabilitation purposes are emerging. There have been a few research studies on the automatic detection of frustration during gameplay [41,42], automatic classification of emotion during gameplay [25,43], engagement during learning [33,44,45] and attention [46,47,48], among others, but only a limited amount of studies exists on the automatic engagement analysis during video gameplay.

Table 1 shows a summary of the most relevant past works. The work of Yun et al. used the cognitive dimension of engagement and focused on gauging concentration (while playing a mobile video game on a smartphone) as a component of engagement using FP1 and FP2 electrodes (FP means frontopolar, a region near the forehead) and three EEG frequency bands, namely the sensorimotor rhythm (SMR) wave, Mid_β wave, and *θ* wave [49]. The index that was used to determine the level of concentration was the addition of the SMR wave and the Mid_β wave divided by the *θ* wave. The system was able to improve the concentration of users. Andujar et al. [50], on the other hand, sought to compare the engagement in learning between the video game mode and the traditional handout mode. They used the Emotiv EPOCX device and Emotiv proprietary EEG engagement scoring algorithm to evaluate the engagement with both the video game and reading modes. The same task was presented in both modes. The video game mode elicited better engagement. However, by policy, Emotiv does not provide access to its engagement algorithm details; hence, it is unknown how the engagement was calculated. Balducci et al. focused on affective ludology, where the emotional dimension of engagement was used in game design to elicit flow and boredom at different game levels. They associated the engagement with attention and participation. Balducci et al. [13] explored affective game design using various game genres and a real-time evaluation of the player’s engagement using the proprietary Emotiv EPOC engagement algorithm to classify the game modes into either boredom or flow levels, and achieved 92% accuracy with SVM.

Ozkul et al. [28] used other physiological signals, such as skin conductance, skin temperature, blood volume, and heart rate, to evaluate the engagement in a video game. The study focused on the behavioural dimension of engagement, using the patient’s performance when reaching the task and physiological measures to evaluate engagement. They wanted to use the estimated engagement of the player to dynamically adjust the difficulty of the game. The study used a partially ordered set master (POSM) and increment/decrement one level (IDOL) algorithms to explore the difficulty adjustment in a computer-based fruit picker game for an upper extremity robot-assisted rehabilitation system.

The work of Ghergulescu and Muntean [51] also employed the Emotiv EPOC and its proprietary EEG engagement algorithm, although the authors acknowledged the existence of brain wave engagement indices. The study used the time on tasks (the duration of time required to complete a task) to model the engagement, arguing that the time taken to complete a task is one of the significant indicators of engagement. They used the behavioural dimension of engagement and assumed that an increase in motivation is associated with an increase in engagement [51]. The time on task as an engagement metric explains up to 76.2% of the variance in engagement change.

When engagement was measured, most of these studies targeted the young adult population and used strictly controlled laboratory settings with many wearable systems, such as EEG headsets with many electrodes, gaze monitoring systems, and cameras, which makes them impractical for home use. The work of Diaz et al. [43] used JINS MEME eyewear and an OpenBCI headset to capture EEG, EOG, and motion data for emotion classification. Ozkul et al. [28] used several biofeedback sensors, such as blood volume pulse, skin temperature, and skin conductance, with a self-assessment manikin (SAM), to measure the emotional engagement and to dynamically adjust difficulty during robot-assisted rehabilitation tasks. Sandhu et al. [52] also used four devices (an EEG device, an eye tracker, a Galvanic skin response device (GSR) and a heart rate variability (HRV) device) to evaluate student engagement in a learning setup.

More recent papers such as Amprimo et al. [53] used a mixture of eye blinks and EEG features to determine the engagement during video gameplay. The study employed an engagement index (*β*/(*θ* + *α*)), besides a concentration index and Event-Related Desynchronisation and Event-Related Synchronisation (ERD/ERS) as engagement features. ERD is linked to engagement and active mental processing, a decreased power in the alpha and beta band, and an increased cortical activity, while ERS is linked to disengagement or a more relaxed brain state, an increase in power in a frequency band, and a reduced cortical activity. Four different models were developed, and discriminant analysis (DA) offered the best performance, at a 98.6% accuracy when classifying the resting and active engagement in the game. The work of Apicella et al. [33] sought to automatically determine student engagement in a learning environment using EEG with an experimental setup that involves a continuous performance test, background music, and social feedback. It adopts the cognitive and emotional components of engagement. They compared the performance of five different strategies, including traditional engagement index (*β*/(*θ* + *α*)), Butterworth–Principal Component Analysis (BPCA), filter bank, domain-adaptation, and Butterworth–Common spatial pattern (BCSP) along with several ML algorithms. SVM offers best result at approximately 77% accuracy, alongside filter bank and common spatial pattern. Natalizio et al. [39] also used a filter bank and engagement index (*β*/(*θ* + *α*)) with a linear discriminant analysis for the real-time engagement estimation using a d2 test, video, and a Tetris game. The models achieved a classification accuracy of 90% for the filter bank and 81% for the engagement index, on average, when tested on an independent d2 test paradigm recording (resting state against engagement state).

In summary, the existing research reveals several gaps. First, the Emotiv EPOC device and its proprietary EEG engagement metric algorithm have been extensively used in engagement studies; however, the algorithm’s formula is often not described. Second, participant ages in these studies primarily range from 12 to 55 years. Third, no studies have specifically investigated automatic engagement detection in older adults during video gameplay. Additionally, very few recent studies have utilised EEG engagement indices (e.g., Amprimo et al. [53]), which indicates a shift from Emotiv proprietary algorithm towards using EEG indices in engagement studies.

This study makes the following novel contributions to this field of study: recruiting older-adult participants, using existing EEG-based engagement indices with limited wearable systems instead of the Emotiv proprietary algorithm to monitor the engagement in video games. The ML potential in an EEG feature-based engagement classification was established for both the younger and older adult populations.

## 3. Materials and Methods

### 3.1. Materials

The materials used for the experiments were a video game, an EEG wearable device, a laptop, a desktop computer, and a flow state scale of occupational task questionnaire. The adjustable seat is placed so that the participant is 1 m away from the screen.

#### 3.1.1. Game and Wearable Device

**Figure 1:**
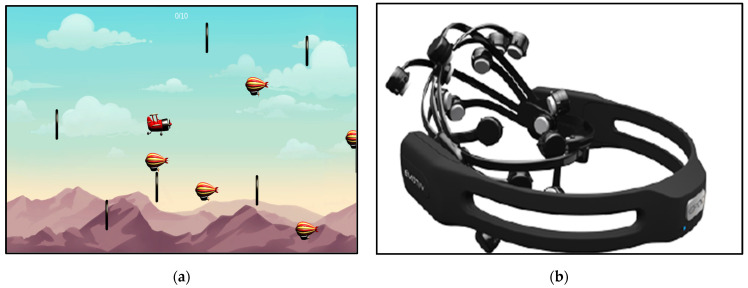
(**a**) Stunt plane game; (**b**) Emotiv EPOCX headset.

A passive BCI experiment was designed using a modified stunt plane game developed by the Glenrose Rehabilitation Hospital. The game depicts a flying plane, with obstacles in the form of striped balloons and targets in the form of black rings, as shown in Figure 1a. The plane is expected to enter or touch the black rings (targets) as much as possible and avoid the striped balloons (obstacles) as much as possible. Most of the games developed at the Glenrose Rehabilitation Hospital were tested to see which ones contain components of cognition, such as executive function, working memory, and inhibition, among others. The selection of the stunt plane game was based on a comprehensive evaluation of various games developed at Glenrose Rehabilitation Hospital for rehabilitation interventions. The stunt plane game was chosen because it was specifically designed for cognitive rehabilitation. Additionally, the game’s rules are simple and easy to under-stand, providing a straightforward, yet sufficiently challenging gameplay experience for both younger and older adults. The game is structured to engage several cognitive processes, including sustained attention (maintaining focus and adhering to the rules until the level is completed), dual-tasking (processing multiple streams of information to navigate through the rings while simultaneously avoiding the obstacles), and processing speed (rapidly interpreting visual stimuli and executing precise motor actions to control the plane). Despite the game’s potential of as a cognitive rehabilitation tool, its effectiveness has not yet been systematically tested. Before evaluating the effectiveness of video or computer game-based interventions on the cognitive function, it is essential to assess how engaging these games are for the target population. Engagement is a critical factor in the success of such interventions, as it influences motivation, adherence, and the overall outcomes. Therefore, this study focuses on exploring the engagement elicited by the stunt plane game in adults and older adults, measured via both subjective and objective engagement metrics.

The setup involves a wearable EEG set called Emotiv EPOCX (EMOTIV, San Francisco, CA, USA). The headset has 14 wet EEG electrodes/channels (saline-based) and two reference points (CMS/DRL references at P3/P4, or a right/left mastoid process as an alternative). The sampling frequency of the device is 128 Hz, and the EEG data are stored in the cloud. Emotiv EPOCX has all the necessary regulatory approvals and takes an average of 20 min to setup (Figure 1b).

#### 3.1.2. Flow State Scale of Occupational Tasks

The flow state scale of occupational tasks developed by Yoshida et al. [19] was used in this study as a self-reported measure of engagement. The scale was reported to have good validity and reliability when measuring the flow state [19]. It is a 14-item Likert scale questionnaire that measures the participants’ subjective level of engagement. The scale has a range of 0–7, where “0” means strongly disagree and “7” means strongly agree; “4”, the middle value, was considered undecided. The dimensions and items measured by the scale include a sense of control, positive emotional experience, and absorption by concentration. The total raw score will sum up to a value between 0 and 98, while the rated score will be between 0 and 7 and can be calculated from Equation (1).

(1) $$rated\;score=\frac{total\;raw\;score\;by\;each\;participants}{14}$$

**Figure 2:**
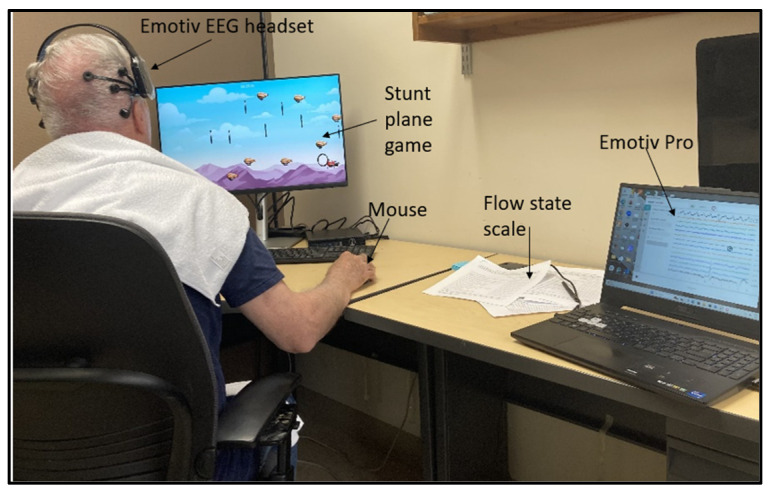
A participant going through the experiment.

In addition, the demographic information of the participants was collected. The experimental procedure and purpose were fully explained to the participants, and written informed consent was obtained from them before they participated in the study. Ethics approval was obtained from the University of Alberta Research Ethics Board with registration No. Pro00142510, 12 June 2024. The setup is as shown in Figure 2.

### 3.2. Design of Experiment

This was a one-time experiment with four stages. The game was designed so that the difficulty level increases from level 1 to level 10; there was a 10% increase in the game speed for every increase in level. It is important to calibrate the player’s skill and to perform calculations on the data recorded to determine the optimal level for each participant. Since individuals tend to have differences in the ability to play games, and what was easy for one person might be hard for the other, it is very important to identify the individuals’ optimal game level based on the empirical data (in-game metrics) rather than choosing an arbitrary general level for everybody.

#### 3.2.1. Performance Index Development

**Figure 3:**
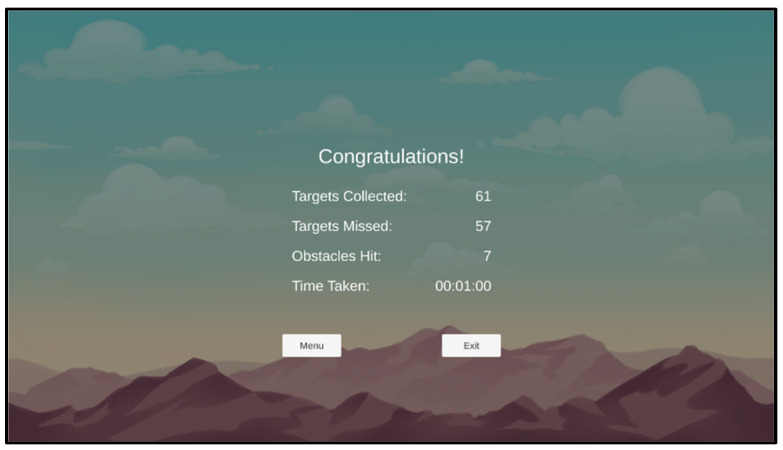
The in-game metrics.

For this study, the following 3 levels of game difficulties were arranged: easy, optimal, and hard. Level 1 was the easiest in the game, and it was assumed to be the easy level for all participants, as the tasks at this level were below the capacity of all of the participants in the pilot study. The in-game metrics were recorded to achieve the optimal level for each participant, and a performance index formula was developed to help calculate the best score and the optimal level across the 10 levels. The three in-game metrics were as follows (see Figure 3):

TC = Total collected items (the number of rings touched or entered)

TM = Total missed items (the number of rings not touched or entered)

OH = Number of obstacle hits (the number of coloured balloons hit)

Using the concept of a direct assignment in a ratio analysis and weighted decision-making for performance modelling [55], weighted coefficients/constants were assigned according to the level of importance of each in-game metric as follows:

TC-assigned 5=α

TM-assigned 5=β

OH-assigned 2=γ

TC and TM were assigned the same weighted value because they contributed equally to the performance of the player. The item missed is the item that should have been collected, and the item collected is the item that could have been missed. Hence, they contributed equally to the performance in the game, and so a weight of five was assigned. The OH effect was limited and did not directly affect TC; however, it was somewhat important, as it measures the player’s dexterity (how well can they collect the target items without hitting the non-target items). Hence, a weight of two was assigned.

By the weighted ratio assignment law [55]:

(2)   α+β+γ=1

However, 5+5+2=12 not 1

Hence, weighted ratio adjustment was performed using the following equation:

(3) $$\frac{\alpha }{\alpha +\beta +\gamma }=\frac{\beta }{\alpha +\beta +\gamma }=\frac{5}{5+5+2}=0.4167$$

(4) $$\frac{\gamma }{\alpha +\beta +\gamma }=\frac{2}{5+5+2}=0.1666$$

The performance Index (PI) was calculated as follows:

(5) $$PI=\frac{1}{\alpha +\beta +\gamma }(\alpha {TC}^{\prime }+\beta {TM}^{\prime }+\gamma {OH}^{\prime })$$

Note that these three parameters (TC, TM, and OH) were not in the same range of scores. Hence, their interaction, as indicated in the PI, would yield a biased result. To avoid this, the in-game metrics TC, TM, and OH were normalised using min–max normalisation, before being used in the PI formula. Hence, the new formula becomes the following:

(6) $$min\_max=\left(\frac{x-min}{max-min}\right)$$

where $\alpha^{\prime },\;\beta^{\prime },\;and\;\gamma^{\prime }\;are\;the\;adjusted\;weights;\;{TC}^{\prime },\;{TM}^{\prime },\;and\;{OH}^{\prime }\;are\;the\;min\;-\;max\;normalized\;values\;of\;in\;-\;game\;metrics$:

The PI was used to obtain a holistic score at each level of gameplay, as it considers all the outputs when determining the final score. For example, the datasheet for one of the participants is shown in Table 2, where the PI at each level of play is calculated using Equation (5). The percentage of the normalised PI was then calculated from the PI score for that participant (using the min–max normalisation of Equation (6)) to know the best performance level relative to other levels. A level corresponding to 100% in the %PI’ column becomes the optimal level, as it indicates the level with the best/highest score for that participant.

#### 3.2.2. Experimental Procedure

**Figure 4:**
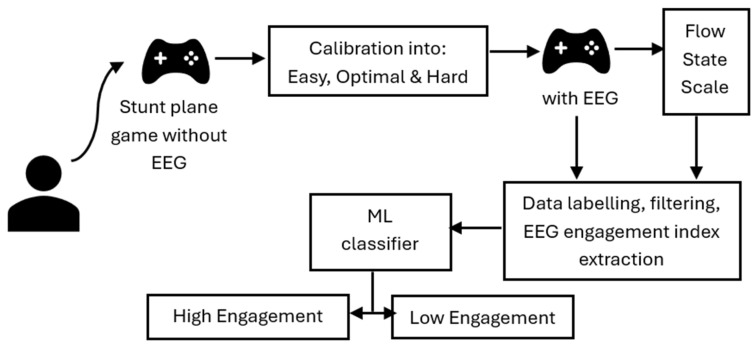
Flow diagram of the procedure.

After an explanation of how to play the game, a written informed consent was obtained from each participant before starting the game. In the first phase, the participant played the game from level 1 to 10 without the EEG headset, and the data obtained were used to calculate the easy, optimal, and hard levels for each participant (see Figure 4). The easy level was level 1 for all of the participants, while the hard level was 4 levels above the calculated optimal level. The participant then played the game again (only at the previously determined easy, optimal, and hard levels) with the EEG headset on for 2 min, resting for 5 min between each gameplay session, and filling out the flow state scale of occupational tasks within that time [32]. This allowed the effect of the previous gameplay session to wash out before the next one began. The reported engagement score and the EEG data were then processed and the rated engagement score from the flow scale was used to label the processed EEG data, which were then fed to the machine learning classifier to be classified into “High” or “Low” engagement.

### 3.3. Signal Preprocessing

The acquired EEG data were sampled at 128 Hz and collected for 2 min. EEG data are very noisy and often contaminated by artefacts such as baseline wander (0–0.5 Hz), powerline noise (50/60 Hz), electromyography noise (high-frequency noise: 20–500 Hz), and electrode/body movement noise [56]. No data interpolation was needed as there was no data loss, and a simple preprocessing pipeline was adopted. During the data collection, the impedance of the electrodes was checked (proper contact of the electrode with the scalp was ensured) and the signal quality index in EmotivPRO live streaming was ensured to be between 90 and 100% before starting data collection. Hence, after collecting the EEG signal, it was denoised using a fourth-order Butterworth band pass filter (0.5 Hz–45 Hz) as implemented in [33]. The band pass filter helped to suppress the high-frequency muscle noise of the scalp and the slow drift (and possible ECG) artefact, which are characterised by a low frequency [57]. The 60 Hz powerline noise (North America standard) has also been eliminated as the upper cut-off frequency of the band pass filter was 45 Hz. The infomax method of independent component analysis (ICA) from the MNE Python library (version 1.5.1-https://mne.tools/1.5/auto_examples/preprocessing/muscle_ica.html, accessed on 12 November 2024) was used to automatically further remove the muscle artefact components. The automatically excluded components were also manually inspected before removal. The analysis was performed in Python environment version 3.11.3. Because of the unavailability of dedicated recording channels for EOG, EMG, and ECG in emotive EPOCX, the commonly employed state-of-the-art methods in the research by Urigüen and Garcia–Zapirain [58] for eye blinks, muscle, and heartbeat removal were not implemented in detail. EEG data were segmented into a one-second non-overlapping window, as implemented in [59]. The rated score of high or low was used to label the segmented EEG data. The self-reported engagement score for the 2 min of playing the game was rated either as high (if the score was ≥4.5 points) or low (if the score was <4.5), as adapted from the flow state scale of occupational tasks. Each segment contains 128 EEG data points. The absolute power of the wave bands was calculated using the multitaper method of power spectral density. The typical features of engagement called engagement indices found in the literature were *β*/(*θ* + *α*), *β*/*α*, and 1/*α*, where Theta [*θ*: 4 Hz–8 Hz], Alpha [*α*: 8 Hz–13 Hz], and Beta [*β*: 13 Hz–30 Hz]. These three EEG wave bands are particularly important, as an increase in the beta wave band power has been linked to an increased brain activity when performing a mental task, while low mental activities were highly correlated with high alpha and theta power [60,61]. EEG engagement indices are the EEG-based metrics used to assess the engagement level [62]. The EEG engagement index 1 (*β*/(*θ* + *α*)) was first proposed by Pope et al. [63] at NASA, while index 2 (*β*/*α*) and index 3 (1/*α*) cannot be linked to anybody in particular, but they have been used in the literature to examine mental engagement and attention [54,62,64]. For these engagement indices, the power of these frequency bands was calculated using power spectral density, and the average band power over the one-second-long EEG data were calculated for each of the 120 segments that made up the 2 min of gameplay. This was repeated for all three engagement indices, 14 channels, and each difficulty level.

#### Data Quality Analysis

Evaluating data quality, especially in commercial EEG devices, is gaining attention [57,65]. The signal-to-noise ratio (SNR) approach in the research by Anders et al. [57] was adopted in this study for the signal quality index, where the signal of interest and noise were to be defined based on the focus of one’s study (i.e., Anders broadly defined the Delta [*δ* < 4 Hz], Theta [*θ*: 4 Hz–8 Hz], Alpha [*α*: 8 Hz–13 Hz], Beta [*β*: 13 Hz–30 Hz], and Gamma bands [*γ*: 31 Hz–100 Hz] as the frequency of interest and high frequency bands as noise (100–125 Hz)). The band-filtered EEG signal was subjected to SNR Equation (7), where the *band power* was the individual signal of interest (theta, alpha, and beta bands) and the *noise power* was the high frequency noise (30–45 Hz muscle noise and 50/60 Hz powerline noise).

(7) $$SNR=10\log_{10}\frac{band\;power}{noise\;power}$$

SNR is measured in decibels (dBs). The signal quality index (SQI) was calculated as the power spectral density of the individual channels averaged across the 2 min windows at the easy, optimal, and hard levels for each participant. The signal power across the individual frequency bands was computed separately and then added to form the total signal power. The noise power was the summation of the total signal power in the defined noise bands. The summarised SNR across the channels is shown in Table 3, and more details can be found in Supplementary Materials.

From Table 3, the SQI ranges from 2.77 dB to 10.42 dB indicated a fairly good signal quality across board. There were no participants with a bad SQI (i.e., with 0 or negative values [57]). 

### 3.4. Participants’ Description

A total of 29 participants were recruited, and 27 of them completed the experiment. The mean age of those who completed the study (n = 27) was 44.77 years (SD = 19.12). There were 18 young adults, with a mean age of 33.77 years (SD = 6.12), and 9 older adults, with a mean age of 70.77 years (SD = 2.85). A total of 65% of the participants were male and 35% were female. Two of the participants dropped out. The descriptive statistics of the participants’ demographics in Table 3 show that trade school was the most common highest level of education among the older adults (55.56%), while a bachelor’s degree was the most common among younger adults (44.44%). There was no elementary school certificate holder among the participants, indicating that they were all educated, but at different levels. The statistics on their residential status showed that most of the participants, both young and older adults, live in a community with others, while very few live alone. None of the participants live in an assisted living facility, residential home, or a university residence.

Table 4 also shows that many older adults and young adults play video games daily. However, older adults tend to play video games for a shorter period (usually between thirty minutes to two hours) than young adults (usually between thirty minutes to four hours and more). In terms of medical conditions, the most common medical condition among the older adults was high blood pressure, while mental health, behavioural, or neurodevelopmental disorders (i.e., anxiety, attention deficit disorder (ADD), post-traumatic stress disorder (PTSD) and attention deficit hyperactivity disorder (ADHD)) were the highest among young adults.

### 3.5. Statistical Analysis

The information obtained from the demographic forms and the flow state scale was analysed using descriptive statistics. This includes age, sex, and level of education, among other things. To answer research question one regarding the differences in the (self-reported) levels of engagement across the game levels, all data were tested for normality using Shapiro–Wilk’s test. Self-reported engagement scores were found to be normally distributed for the easy and hard levels, but non-normal for the optimal levels; therefore, a Friedman test of the related samples was implemented to assess the difference in the distribution of the self-reported engagement scores across the easy, optimal and hard game levels. A post hoc test was performed at a 0.05 significance level with a Bonferroni correction for multiple tests, which helped to prevent false positives/type 1 errors when dealing with multiple comparisons by adjusting the significance level.

To answer research question two asking how the EEG engagement indices vary across the game’s difficulty levels, a Friedman’s test of the related samples was conducted to identify any differences in the EEG-based engagement index score across the game difficulty levels, as the data were not normally distributed. This was repeated for the EEG data for engagement index 1, 2, and 3 ([EEG index 1 = *β*/(*θ* + *α*), EEG index 2 = *β*/*α*, and EEG index 3 = 1/*α*]).

### 3.6. Machine Learning Analysis

**Figure 5:**
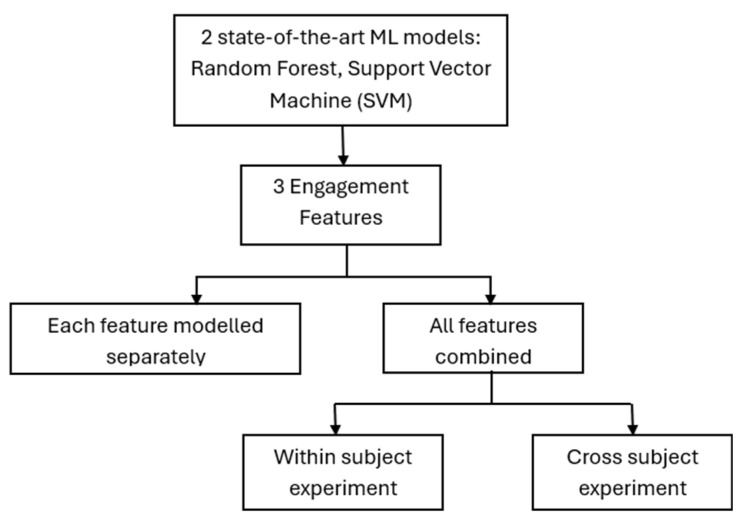
ML workflow.

A machine learning approach was used to answer research question three, asking how accurately can self-reported engagement scores be classified using EEG data. The machine learning strategy adopted in this study included within-subject and cross-subject classifications. Two ML classifiers were used: random forest (RF) and support vector machines (SVM). Random forest (RF) is an ensemble decision tree classifier, based on supervised ML, which uses a collection of tree predictors, where each tree is assigned an independent vector sample randomly with an identical distribution [66]. SVM, on the other hand, is a binary classifier that uses a decision hyperplane to differentiate between the data by finding the hyperplane with the maximum separation from the margin of classes [33]. The classifiers were selected based on their record of excellence in EEG data classification in the literature [33,43,67]. The workflow of the ML strategy is shown in Figure 5.

The data were split as follows: 80% was used for training and 20% for testing, as is standard practice. A 10-fold cross-validation was used when each EEG feature was modelled separately, while a 5-fold cross-validation was used for the whole data/cross-subject classification with all of the EEG index features used (to reduce computation time). A nested cross-validation with an outer layer of five and an inner layer of three was used for the within-subject classification, with all of the EEG index features combined. The data were normalised using a standard scaler function to bring them under the same scale of values, so that each feature’s contribution could be detected without a bias. Hyperparameter tuning was adopted to help optimise the classification results so that the best parameter was chosen for the algorithm (see Table 5). The data used for the machine learning is summarised in Table 6. Out of the 27 participants, 11 participants’ data were not used in the ML algorithm, as they did not provide self-reported engagement scores clearly above or below the set threshold of “High” and “Low”, according to the flow state scale of occupational task, i.e., neutral engagement (score of four). There were 5760 data points or epochs for each channel, and we had 14 EEG channels.

The performance of the model was evaluated using accuracy and F1 scores, although, for this study, the F1 score better captured the true performance of the model, as the data were imbalanced. Since most papers report accuracy alone, we reported both accuracy and the F1 score to allow for a comparison with other studies. It is worth noting that for clinical applications an accuracy above 80% is assumed to be acceptable [59,68].

## 4. Results

### 4.1. Reported Engagement Score Across Game Levels (RQ1)

**Figure 6:**
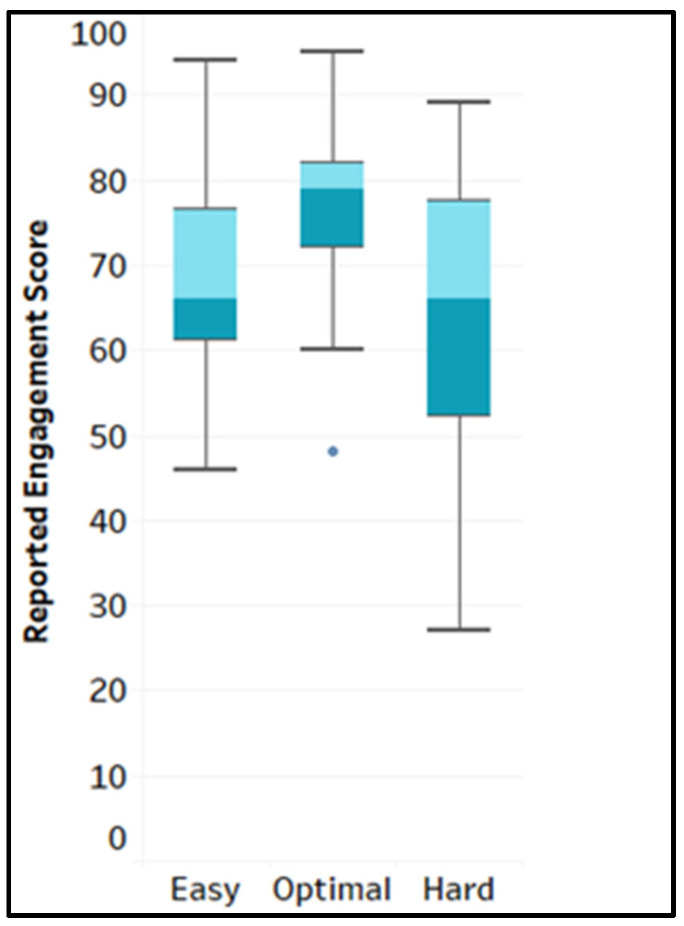
Box plot of the reported engagement score. Note: “**.**” is outlier.

To answer the research question 1, “Are there differences in the levels of engagement (self-reported) across game levels?”, the self-reported engagement score was extracted from the flow state scale of the occupational task questionnaire across the different game difficulty levels (easy, optimal and hard). The mean (M) and standard deviation (SD) of the flow state scale score were as follows: easy (M = 4.91, SD = 0.81), optimal (M = 5.51, SD = 0.65), and hard (M = 4.59, SD = 1.15). The raw engagement score was tested for normality before choosing the correct statistical approach. Following the Shapiro–Wilks test, the reported engagement score for each difficulty level was normally distributed (*p* > 0.05) for the easy and hard levels, but not-normally distributed for the optimal level (*p* < 0.05). Using SPSS software version 29.0.1.0, a related sample of Friedman’s two-way analysis of variance by ranks was conducted to determine the effect of the different game difficulty levels on the reported engagement scores. The values 1.87, 2.41, and 1.72 were the mean ranks of the reported score for the game’s easy, optimal and hard levels, respectively. The main effect of the game levels was significant, with χ^2^(2, N = 27) = 7.22, *p* = 0.027. This was followed up by a post hoc test of comparison using the Bonferroni correction, which showed that the reported engagement score (subjective score) was statistically significantly higher at the optimal level than the hard level (*p* < 0.035), but not significantly different from the easy level (*p* < 0.145), even though it had a higher mean rank. However, the reported engagement score distribution was not significantly different across the easy and hard levels (*p* = 1.000). Figure 6 shows the boxplot of the distribution.

### 4.2. EEG Engagement Indices Across Game Levels (RQ2)

The average EEG indices were obtained from the band power of the EEG signal across the 14 channels. The EEG indices were not normally distributed across the game levels (*p* < 0.05), and a related sample of Friedman’s two-way analysis of variance (ANOVA) was conducted for each of the three EEG engagement indices, as shown in Table 7.

**Figure 7:**
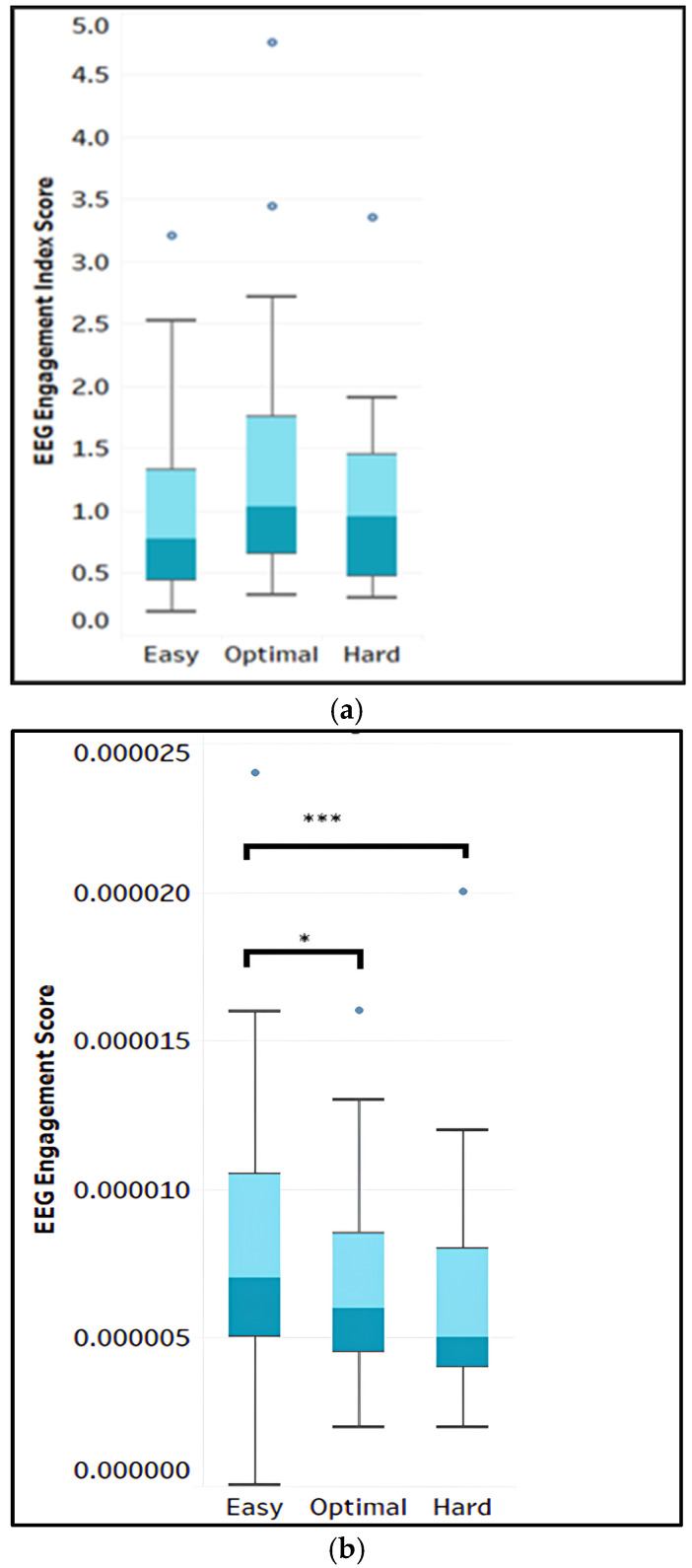
Box plot of the EEG engagement score in (**a**) index 1 and (**b**) index 3. [*p* < 0.001: ***, *p* < 0.05: *] Note: “**.**” is outlier.

The Friedman’s test on the main effect of the game difficulty level on EEG engagement indices 1 and 2 was not statistically significant. However, EEG index 3 revealed a significant main effect of the game difficulty level on the engagement score, with χ^2^(2, N = 27) = 24.16, *p* < 0.001. The mean ranks were 2.65, 1.91, and 1.44 for the easy, optimal and hard levels, respectively. A post hoc test of multiple comparison with a Bonferroni correction was implemented. The pairwise comparison showed that the EEG index 3 score at the easy level was significantly higher than at the optimal level (*p* = 0.019) and the hard level (*p* = 0.000), respectively (see Figure 7). In contrast, the mean rank of EEG index 3 scores did not significantly differ between the optimal and hard levels (*p* = 0.267).

### 4.3. Machine Learning Analysis (RQ3)

To answer research question 3, the machine learning algorithms were developed using two state-of-the-art ML classifiers in an EEG analysis: Random Forest (RF) and Support Vector Machine (SVM). For each EEG engagement index as an input feature, the RF and SVM models were developed to classify the engagement in the video game into “High” or “Low”. Based on the common practice of EEG data analysis in rehabilitation, a within-subject model classification was implemented, as shown in Table 8, and the mean accuracy and F1 score were estimated. Then, another model, which combined the three EEG engagement indices as input features, was tested. Combining the engagement indices simply means that the columns of EEG index 1, 2, and 3 data were used together to predict the engagement.

**Figure 8:**
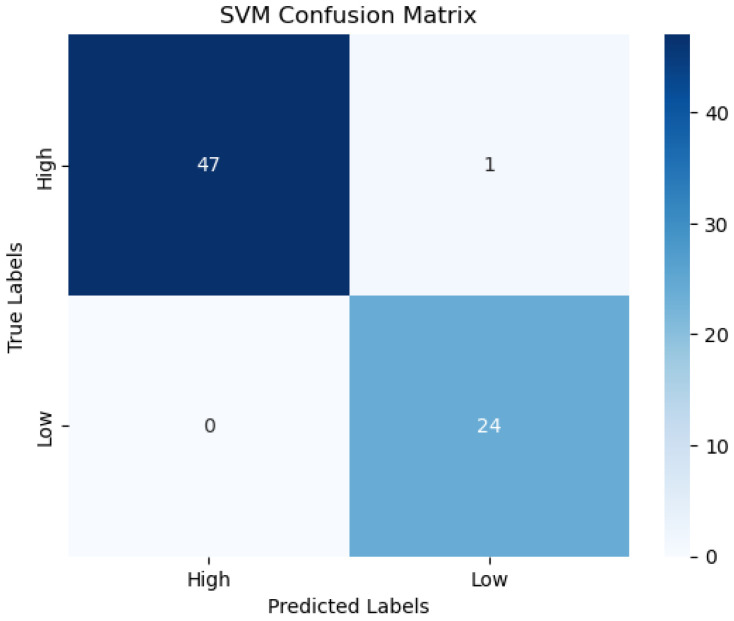
Confusion matrix for #1 SVM model under EEG indices combined.

Table 8 reveals that the combined feature model had the best average accuracy and F1 score (89% and 86%, respectively), followed by the EEG index 3 model (accuracy = 83% and F1 score = 78%). These values exceed the threshold of 80% required for satisfactory performance in rehabilitation applications [59]. A similar table was developed for the SVM classifier (see Table 9). Figure 8 also showed the confusion matrix for #1 under the SVM classifier, with a class imbalance of 48 and 24 for high engagement and low engagement, respectively.

A cross-subject study was also implemented to see how the models would perform if all the participants’ data were pooled together and run as one ML model instead of within-subject/personalised classification. Only the combined feature method is used here, as it offers the best performance in the within-subject tests, as shown in Table 10. The table revealed the highest score of 81% accuracy and 80% F1 score obtained from the SVM classifier. This is a very good result for a cross-subject classification, as it reached the 80% benchmark for satisfactory classification performance for studies in the rehabilitation field.

**Figure 9:**
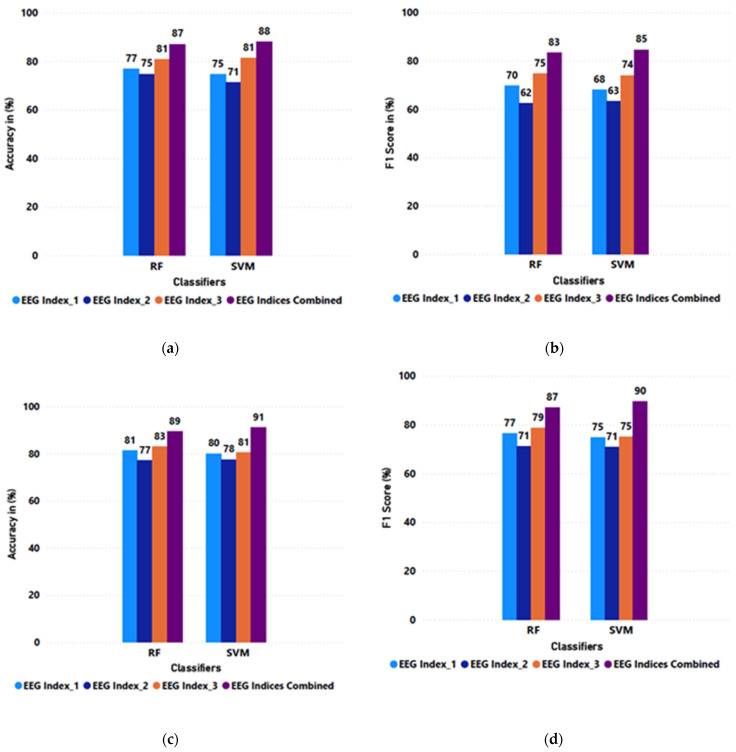
(**a**) Average accuracy across EEG indices and combined features for older adults (60 years and older), (**b**) average F1 score across EEG indices and combined features for older adults (60 years and older), (**c**) average accuracy across EEG indices and combined features for young adults (18–59 years), (**d**) average F1 Score across EEG indices and combined features for young adults (18–59 years).

Since the study includes two population categories, the average performance of EEG indices in classifying the engagement across these two population samples was examined to answer research question 3.1, “Do the older adults’ and young adults’ EEG data compare with each other in terms of performance reports?” The ML included thirteen young adults and three older adults. The bar plot of the average performances across the EEG indices based on age group is shown in Figure 9. The plots showed that the F1 score for the young adults’ and older adults’ classification results was above 80% in the combined feature model. However, young adults’ performance report (F1 score) of 90% was better than the older adults’ report of 85%.

**Figure 10:**
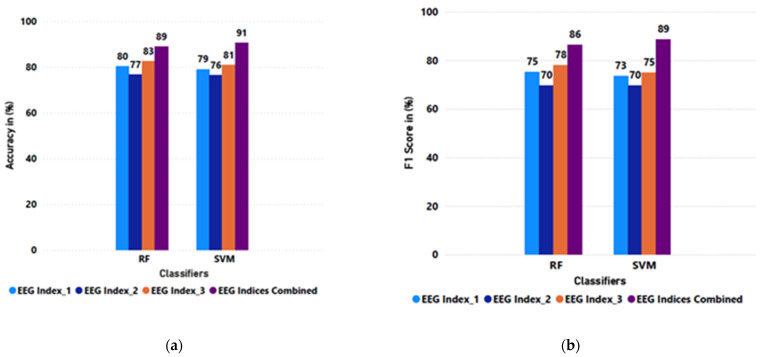
Histogram of (**a**) average accuracy and (**b**) average F1 score for all participants in within-subject classification.

To answer research question 3.2, “Which classifier is better in performance”?, clustered column charts of RF vs. SVM were plotted, as shown in Figure 10. The plots showed that the combined EEG indices model offers the best performance, and the SVM is consistently the best classifier at a 91% accuracy and 89% F1 score. The cross-subject performance report in Table 8 also supports this claim, as the SVM had the best score at an 81% accuracy and an 80% F1 score.

## 5. Discussion

The demographic data showed that older adults were predominantly trade school graduates. This observation is not unexpected, as there were fewer higher education institutions and less drive for a high formal education during their youth [69]. Most of the participants in both of the age brackets lived in a community with others, indicating that they were a virtually healthy population, while none lived in a care home. Playing video games daily was found to be common in both age brackets, which further shows the potential acceptability of implementing video game-based therapy for these populations. Incorporating therapy into a system/programme that people are already familiar with often helps enhance the acceptability and usability [70,71]. Although both young and older adults played video games, the report shows that older adults played for a short period of time, which could affect the efficacy of video games as a therapeutic intervention to delay the cognitive decline. Hence, ways of enhancing engagement should be sought to increase playtime. High blood pressure was a common comorbidity among the older adults [72]. The report in Table 3 confirms this; it shows that high blood pressure was the most prevalent health condition in older adults, while mental, behavioural, or neurodevelopmental disorders, especially ADHD, were the most prevalent health conditions in young adults.

The result obtained when answering research question 1 indicated that changes in the game difficulty levels, from easy to optimal and hard, had significant effects on the reported engagement score. In essence, the participants were more likely to be highly engaged during the game at the optimal level rather than at the easy and hard levels. This could be linked to the fact that the optimal level was calculated individually, and it was selected based on the level that the participants performed best at, so that the difficulty of the task was slightly higher than the skill of the players, but it was still within their capacity. This created a state of flow, which is characterised by high engagement [73]. This observation is in line with the study by Katahira et al. [74], who also used a flow state questionnaire and EEG data with a computerised arithmetic task at three levels (boredom, flow and overload). The highest subjective ratings were observed during the flow state, when examined on an item-by-item basis, specifically on items designed to measure engagement [74].

Even though the mean rank of engagement at the optimal level was higher than at the easy level in the current study, it is not statistically significant. This plausibly means that the easy and optimal levels offer similar feelings of “High” engagement, while the hard level is more attributed to “Low” engagement. This can be explained by the large proportion of older adults who reported “High” engagement at the easy level, perhaps due to an age-related decline in their cognitive skills, such as processing speed, compared to young adults [75]. It is possible that the easiest level was not that easy for the older adults, meaning that they did not feel bored and unchallenged, but instead challenged in some way. Processing speed declines with age, so the easiest level of the game could be challenging for the older adults but not for the young adults. In fact, many older adults reported “High” engagement across all three game difficulty levels.

The results from research question 2 indicated that the EEG engagement index 1 (*β*/(*θ* + *α*)) and EEG engagement index 2 (*β*/*α*) did not have a significant main effect on game difficulty levels. This means that even though the EEG engagement score was higher at the optimal level, as shown in Figure 7a, it is not statistically different from the scores at the easy and hard levels. This lack of significance could occur due to the small sample size, as well as due to the combination of several frequency band powers and the use of data from several brain regions at once (frontal, temporal, occipital, and some parietal regions). In addition, it could be that a more complex non-linear relationship existed between the EEG engagement score and the game difficulty levels [76]. However, EEG engagement index 3 (1/*α*) had the following significant effect: the EEG inverse alpha band power was significantly higher at the easy level than at the optimal and hard levels. The amplitude of the inverse alpha power continued to increase as we moved from the easy to the hard level; this aligns with the previous studies [54,74], which have established that alpha power tends to be a significant index for task difficulty and task load on the working memory. The higher the difficulty of the task, the lower the alpha power/amplitude. Hence, the inverse alpha power used as the engagement indices in this study increased as the task difficulty increased from the easy to the hard level.

The research question 3 results revealed that the combined feature set model was the best model, with an accuracy of 91% and an F1 score of 89%. This is followed by the EEG index 3, with an 81% and 75% accuracy and F1 score, respectively. This is consistent with the RF table (Table 7), where the combined feature set also offers the best performance in terms of both accuracy and F1 score, and EEG index 3 was the second best. These results also show that each of the EEG engagement indices were very competitive and represented the different key features of engagement. However, combining the three EEG indices (features) offers the best performance, indicating a more extensive capture of the engagement dynamics.

EEG index 1, the most popular EEG engagement index in the literature [33,62,76,77,78,79], was not the best in terms of performance. This result aligns with a few recent studies [33,39], which also found it suboptimal. The performance of EEG index 1 could be attributed to the variation in beta band modulation across the different brain regions, being higher in the frontotemporal regions than in the parieto-occipital regions [39]. Out of all of these studies, only the works of Apicella et al. [33] and Coelli et al. [62] used a game-like continuous performance task (CPT), while Berka et al. [76] and Nuamah et al. [79] used several mental tasks, such as arithmetic tasks, geometric figure rotation, multi-level forward/backward-digit-span, and a memory test, among others. Other researchers focused on audio engagement during lectures [77] and attention measurements during real-life work environments [78].

The studies by refs. [54,64] explored EEG index 2 as a feature of engagement in games, but this feature was not investigated in machine learning applications. Hence, there is limited prior knowledge about its effectiveness as an ML feature in EEG engagement studies. The work of Kislov et al. [64] employed a neuromarketing paradigm, where the participants were made to watch coloured banner ads on a computer while their EEG and eye-tracking data were captured. Coelli et al. [54] adopted a continuous performance task (CPT) for sustained attention.

EEG index 3 has been featured in very few studies such as [54], where it was used as an indicator of engagement during a sustained-attention continuous performance test (CPT) experiment, and in the work by Sandhu et al. [52], where it was used as one of the EEG engagement indices in an ML classification of engagement in a reading/learning task. However, in our study, EEG index 3 performed better than index 1 and index 2 as an engagement feature. This could be attributed to the alpha band’s ability to detect variations in workload/task difficulty; in addition, it is the only EEG engagement feature with statistical significance across the different game difficulty levels. However, indexes 1 and 2 were more aligned with the flow theory. Maybe the low sample size contributed to the insignificant difference in EEG index 1 and 2 across the different game difficulty levels, as EEG index 1 was close to being significant at *p* = 0.060.

Research Question 3.1 results showed that young adults’ ML performance reports were better than older adults’ ML reports (5.88% increase in F1 score) suggesting that older adults’ EEG data may compare favourably to young adults’ EEG data when classifying engagement during video gameplay if there were to be larger number of older adults’ data. The plots revealed that both the accuracy and the F1 score of young adults were higher than those of the older adults. The F1 scores from the combined feature model were 90% and 85% for young and older adults, respectively. This can be attributed to the quality of the EEG signals acquired from the participants. López-Loeza et al. [80] have reported significant differences in EEG power between young adults (18–25 years) and mature adults (46–65 years), with a relatively higher absolute theta power and lower Gamma power during learning in the temporal and frontal brain regions for the young adults. More studies, like the work of Ma et al., expressed that there is a relatively lower complexity (randomness and regularity) in the older adults’ resting state EEG data when compared to the young adults [81]. Hence, the lower performance score for the older adults could be linked to the ageing brain. However, this interpretation should be treated with caution due to the low number of older adults in this study. Future research should include further studies with larger sample sizes to provide more conclusive results.

In addition, the F1 scores of 85% and 90% for the older and young adults, respectively, suggest that EEG data can be used to satisfactorily classify the engagement in video games for both age groups, as these performance metrics are above the clinical threshold of 80% [59].

The research question 3.2 results clearly revealed that SVM is the best classifier, at a 91% accuracy and an 89% F1 score for within-subject and 81% accuracy and 80% F1 score for cross-subject performance. This is consistent with the work of many studies on EEG that also find SVM to have the best performance [29,33,82]. SVM has also been found to competitively perform as the second best classifier in the work of Apicella et al. [59], where EEG was used for distraction detection in motor rehabilitation training, as well as in the research by Amprimo et al. [53], where it was used for the binary classification of active gameplay and resting states. The success of SVM in EEG data can be attributed to its high dimensionality and ability to work well with small data sets, which is often the case with EEG data [83]. In addition, EEG often exhibits non-linear characteristics that the radial basis function (RBF) kernel of SVM can capture, and it is robust to overfitting [84].

The demographic information of the 11 participants whose data were not usable in the ML implementation was examined to find a group difference or a pattern that could be used to explain the reason behind their perceived engagement report. However, no clear/substantial pattern was found, except the fact that most of them were older adults. A robust screening of the pre-existing mental health conditions and how they may influence the self-reported engagement score could be explored in future research. The engagement scoring was performed using the flow state scale of occupational tasks and can be used to evaluate engagement in other games. However, the performance index was developed specifically for the stunt plane game.

### 5.1. Clinical Implications

Studies have projected an increase in the number of older adults not only in Canada, but in the world at large [1]. Systems and technologies aiming at improving dementia care are taking the forefront of research, and landmark studies by the Alzheimer Society of Canada have stressed the potential use of cognitive video games in delaying cognitive decline [5]. The findings from our study have shown that older adults engage in video gameplay, and that the games can be adapted for cognitive rehabilitation by using computerised cognitive games, which older adults appear to prefer over the traditional methods [85]. The ML results have shown that older adults’ EEG data can be used in ML to determine their engagement during video gameplay. With more research, the system could be used by occupational therapists and other health care professionals to determine the engagement in older adults or in individuals who cannot report on their perceived engagement, due to reasons such as cognitive decline or motor impairment, that can affect the production of recognisable facial expressions or motor behaviours.

Since engagement is vital for effective rehabilitation, the developed ML algorithm can be incorporated into computerised cognitive games to passively monitor the users’ engagement in real-time and to adaptively adjust the game parameters in order to sustain engagement. Increased engagement can facilitate reaching the dosage of game-based cognitive interventions in other to improve its effectiveness.

### 5.2. Limitation

Even though the results in this study were promising, they need to be interpreted with caution because of several reasons. First, the small sample size limits the generalizability of the findings. Second, our sample consisted of more young adults than older adults; hence, having either an equal number or more older adults in the sample population would provide more validity for the results. Out of the 16 participants used in the ML implementation, 13 were young adults and only 3 were older adults. Third, no standard test was used in this study, as there is no standard test or game for eliciting engagement, as there are for emotions (e.g., International Affective Picture System and Montreal Affective Voice) and attention (e.g., Continuous Performance test, N-Back test, and the Attentional Blink task, among others). In addition, the standard tests, such as the continuous performance test, often adopted in the literature on engagement studies, are only used to test whether there is engagement or no engagement, not differentiate between high or low engagement. In addition, the data were not balanced (that is, the proportion of high- and low-engagement-labelled EEG data was not the same, as we had more labels with “high” engagement than with “low” engagement); hence, the reporting of accuracy as a performance measure, which is the usual way, would not be appropriate (when used for balanced data); the F1 score was added to the performance report as it provides a more objective metric in this case, as it is a harmonic ratio of the precision and recall score. Finally, only EEG data were used to evaluate engagement in this study, and a limited, but satisfactory accuracy was obtained. Hence, incorporating other physiological data like EOG, ECG, or skin impedance may provide other indicators of engagement that can either improve the performance report or help to understand the EEG results.

### 5.3. Recommendations for Future Research

This study used all 14 channel electrodes of Emotiv EPOCX in the classification algorithm from different brain regions, such as the frontal, temporal, parietal and occipital regions. Dividing these channels based on the brain regions and retraining the models could help identify the brain regions that possess most of the information about EEG engagement. Electrode design can then target only those regions which would help in the advancement of the current research on the adoption of a reduced number of electrodes (sparse electrodes) in EEG research and developments. Recently, there has been an increasing number of studies reporting the potential of the EEG gamma wave band as a significant EEG correlate of engagement [86,87]. For example, the study by Halderman et al. [87] divided the gamma waves into low gamma: 28 Hz–44 Hz, medium gamma: 44 Hz–90 Hz, and high gamma: 90 Hz–150 Hz and discovered that the band power in all the wave bands increases as the engagement increases. Studies can be directed towards developing a more comprehensive engagement index that would include gamma wave bands in the formula, since more and more studies are finding the standard engagement indices suboptimal. The primary focus of this study was not to identify new engagement correlates, but to utilise the existing engagement indices with components that have been previously established in the literature as correlating with engagement. Since the gamma wave has not been used in the literature to develop an engagement index (expression or formula), it was not included in this study.

Many older adults reported high engagement scores at the easy level of the adopted stunt plane video game. The set parameters for the easy level, such as the number of obstacles, can be reduced in future work. Lastly, dry electrode EEG devices can be tested; the wet electrodes were inconvenient because they left the participants’ hair moist. Some of the participants in this study found the wet electrodes inconvenient, especially people with thick or large volumes of hair, as more saline solution was needed for an effective electrode connection to the scalp. The methodology can be repeated using a game that older adults used to use, and one they are not used to playing, to check for consistency and possible changes in results.

## 6. Conclusions

This study examined the effects of changes in the game difficulty level on the reported engagement score and the EEG data acquired during gameplay. The acquired EEG data were processed, and the EEG engagement features were extracted for use in a ML classification algorithm to classify the engagement as either high or low. The data showed a statistically significant main effect of the game levels on the reported engagement scores (*p* < 0.027). The study showed that the game’s difficulty level setup elicited higher reported engagement at the optimal level than at the easy and hard levels.

The EEG signal was filtered with a fourth-order Butterworth bandpass filter with a range of 0.5 Hz–45 Hz. The average band power of the Theta, Alpha and Beta frequency bands was used to calculate the engagement indices 1(*β*/(*θ* + *α*)), 2 (*β*/*α*) and 3 (1/*α*) for each channel. The statistical tests showed that the game difficulty levels did not have an effect on the EEG indices 1 and 2, as their mean scores were not significantly different across the game levels. However, the game difficulty levels have a significant main effect on EEG index 3 (*p* < 0.001). The best classifier for the EEG engagement classification was the SVM, for both within-subject and cross-subject models, while the model that combined all the EEG engagement indices as an input feature performed the best. The second-best model used EEG index 3 as an input feature. The EEG engagement index 1 was the third-best model, while EEG index 2 had the lowest performance. The cross-subject model, trained on data from all participants, performed competitively with the within-subject models, achieving F1 scores of 80% and 89%, respectively. This result indicates a high potential for adopting a generalizable model for EEG engagement applications compared to the personalised models that are common in EEG ML studies. Personalised models often offer a better performance, but they are usually computationally expensive.

## Figures and Tables

**Table 1:** Summary of most relevant past works.

Subcategories	Game	Biomarker/Feature	Population/Age	Study	Year
EEG	Smart phone game app	Concentration index	Not specified	Yun Seo et al. [49]	2018
EEG	Video game and handout	Emotiv’s proprietary engagement algorithm	32 participants, no age record	Andujar et al. [50]	2013
EEG	Neverwinter Nights 2	Emotiv’s proprietary engagement algorithm	19 students, no age record	Balducci et al. [13]	2017
EEG	Fire Protocol game	Emotiv’s proprietary engagement algorithm	50 participants, 18–55 years Average: 27 ± 7	Ghergulescu & Muntean [51]	2016
EEG+Eye tracker + Motion tracker	Whack-a-Mole game	PSD, standard deviation (Std) of bands, Delta/Theta, Delta/Alpha, Delta/Beta, Theta/Alpha, Theta/Beta, and Alpha/Beta.	21 participants, >18 years	Diaz et al. [43]	2021
EEG + HRV + GSR+ Eye tracker	Conjunctive Continuous Performance Test	β/(θ+α), β/α, and 1/α absolute band power, Interbeat (R-R) Interval, eye motion velocity	10 participants, 17–22 years	Sandhu et al. [52]	2017
EEG	Grab-Drag-Drop Exergame	Concentration index, Engagement index (β/(θ+α)) and event-related desynchronization/synchronisation	50 participants, 26 ± 4.5 years	Amprimo et al. [53]	2023
EEG	Continuous Performance Test	Engagement index (β/(θ+α)), Filter banks, Domain adaptation, Butterworth Principal Component Analysis	21 participants, 23.7 ± 4.1 years	Apicella et al. [33]	2022
Non physiological signal	Multimodal Game Frustration	Audiovisual data	67 students, 12–16 years	Fuente et al. [42]	2023
Other physiological signal	Fruit picking game	blood volume pulse, skin temperature, and skin conductance	20–37 years	Ozkul et al. [28]	2019
EEG	continuous performance test	Engagement index (β/α)	9 participants, 18–28	Coelli et al. [54]	2015
EEG	D2 test, video and Tetris game	Filter bank and Engagement index (β/(θ+α))	23 participants, 34 ± 7 years	Natalizio et al. [39]	2024

**Table 2:** Optimal level calculation from a participant data sheet.

Level	TC	TM	OH	${TC}^{\prime }$	${TM}^{\prime }$	${OH}^{\prime }$	PI	$\%{PI}^{\prime }$
1	29	0	3	0	0	0	0	51.21027
2	54	6	3	0.245098	0.037267	0	0.086603	77.83074
3	76	10	8	0.460784	0.062112	0.092593	0.150701	97.5334
4	91	27	11	0.607843	0.167702	0.148148	0.158725	100
5	98	44	20	0.676471	0.273292	0.314815	0.115556	86.73052
6	115	57	21	0.843137	0.354037	0.333333	0.148275	96.7876
7	115	89	31	0.843137	0.552795	0.518519	0.0346	61.8459
8	111	119	34	0.803922	0.73913	0.574074	−0.06864	30.11069
9	128	139	42	0.970588	0.863354	0.722222	−0.07564	27.9604
10	131	161	57	1	1	1	−0.1666	0

**Table 3:** Overview of SQI expressed as SNR (dB) across channels.

S/N	AF3	F7	F3	FC5	T7	P7	O1	O2	P8	T8	FC6	F4	F8	AF4	AVG EEG (dB)
#1	10.23	8.57	10.44	8.54	4.86	6.63	7.51	8.98	10.97	11.90	13.57	10.66	13.27	14.37	**10.04**
#2	13.11	9.98	11.08	7.19	6.80	6.89	9.23	9.20	7.74	5.88	11.31	14.36	12.47	14.23	9.96
#3	10.57	4.31	6.20	2.92	6.94	6.12	5.78	5.49	5.61	4.53	5.41	6.16	7.43	13.58	6.50
#4	9.94	9.89	9.43	9.59	10.20	10.47	11.21	11.16	10.48	8.89	10.51	11.65	11.61	10.78	**10.42**
#5	7.69	4.38	3.78	3.00	2.18	1.84	2.82	3.29	2.60	2.82	3.71	3.85	7.13	5.29	3.88
#6	6.68	4.46	4.22	3.12	1.68	1.71	2.40	2.91	3.59	8.21	3.97	4.25	6.61	5.54	4.24
#7	11.39	9.39	12.51	9.57	5.26	6.79	9.04	9.35	5.82	9.93	11.95	12.59	12.59	12.51	9.91
#8	4.94	4.10	4.88	3.95	2.24	3.36	3.76	3.60	3.17	3.87	4.87	4.38	6.30	6.24	4.26
#9	16.50	9.34	12.37	8.21	4.16	5.60	7.09	7.80	8.17	6.87	13.25	13.03	12.28	16.19	**10.06**
#10	5.37	5.56	4.98	4.02	14.88	3.13	2.98	4.20	3.25	5.56	5.43	4.99	9.17	6.00	5.68
#11	9.72	8.97	11.29	7.82	2.93	5.87	7.89	8.35	7.39	4.49	10.76	10.94	11.62	10.15	8.44
#12	9.97	4.63	6.95	4.47	4.96	4.56	6.87	6.93	5.70	6.73	5.62	7.06	6.99	5.60	6.22
#13	6.01	8.97	8.62	6.77	4.30	5.37	8.02	8.18	7.86	5.89	7.87	8.23	7.03	7.39	7.18
#14	5.08	11.55	10.27	11.00	7.60	6.23	8.61	9.73	9.51	7.22	10.00	9.92	10.24	6.20	8.80
#15	10.10	10.28	5.32	7.52	2.33	2.32	2.12	2.97	3.07	3.61	7.81	4.70	14.54	7.64	6.02
#16	6.70	3.54	3.31	4.47	0.07	1.33	2.65	3.33	1.96	0.65	2.65	2.68	4.86	5.06	3.09
#17	6.85	8.16	5.57	5.32	2.73	5.54	6.38	6.23	6.14	4.75	6.96	6.59	9.82	8.41	6.39
#18	6.19	6.45	7.17	6.30	5.83	6.76	8.01	8.40	6.31	5.28	6.47	6.29	6.43	5.69	6.54
#19	4.93	4.30	6.43	3.34	2.44	4.26	4.40	5.76	5.74	1.80	5.94	6.85	7.58	4.19	4.85
#20	10.18	9.04	10.36	8.75	9.21	8.45	9.40	9.86	9.23	10.00	10.52	11.10	11.01	10.97	9.86
#21	8.53	6.67	7.72	5.03	5.53	6.70	7.69	7.02	6.53	5.87	8.47	9.74	10.83	11.46	7.70
#22	3.94	3.83	3.74	2.55	3.92	1.04	2.99	3.08	2.82	3.83	4.51	3.58	5.84	6.35	3.72
#23	4.41	5.53	4.49	2.78	1.53	1.40	1.52	2.05	2.59	2.31	3.72	2.70	9.97	4.50	3.54
#24	3.82	1.82	3.54	2.29	1.52	2.24	3.21	3.17	1.75	2.27	3.17	3.18	3.13	3.74	*2.77*
#25	8.36	10.57	7.66	8.90	9.41	6.49	7.88	8.08	8.39	7.43	9.31	8.30	11.17	11.84	8.84
#26	3.43	2.12	2.68	0.97	2.85	1.27	2.09	2.33	2.35	2.96	3.25	2.88	5.02	5.03	*2.80*
#27	5.52	7.29	6.49	4.25	5.14	3.39	5.92	5.55	3.56	4.15	7.08	6.44	8.78	5.48	5.65

Note: Values below 3 dB are italicised and values above 10 dB are bold.

**Table 4:** Demographic data summary.

Variables		n = 27		Young Adults = 18 (10 Men and 8 Women)		Older Adults = 9 (5 Men and 4 Women)	
Age (Mean, SD)		44.77, 19.12		33.77, 6.12		70.77, 2.85	
Sex (% Male)		65%		55.55%		55.55%	
		n	%	n	%	n	%
Highest degree/Education	Elementary	0	0.00	0	0.00	0	0.00
	Secondary school	0	0.00	0	0.00	0	0.00
	High school	2	7.40	2	11.11	0	0.00
	Trade school	5	18.52	0	0.00	5	55.56
	Bachelor’s degree	11	40.74	8	44.44	3	33.33
	Master’s degree	8	29.63	7	38.89	1	11.11
	MD, PhD or higher	1	3.70	1	5.56	0	0.00
Residential status	LCA	3	11.11	2	11.11	1	11.11
	LCWO	24	88.89	16	88.89	8	88.89
	RH/AL	0	0.00	0	0.00	0	0.00
	UR	0	0.00	0	0.00	0	0.00
	Others	0	0.00	0	0.00	0	0.00
Experience with video games	Daily	9	33.33	5	27.78	4	44.44
	Twice weekly	4	14.81	4	22.22	0	0.00
	Weekly	0	0.00	0	0.00	0	0.00
	1-2X/month	0	0.00	0	0.00	0	0.00
	<once/month	4	14.81	4	22.22	0	0.00
	Few times/year	6	22.22	3	16.67	3	33.33
	Never	4	14.81	2	11.11	2	22.22
Duration of video gameplay	<30 min	6	22.22	4	22.22	2	22.20
	30 min–1 h	11	40.74	7	38.89	4	44.44
	1–2 h	2	7.41	1	5.56	1	11.11
	2–3 h	3	11.11	3	16.67	0	0.00
	≥4 h	3	11.11	3	16.67	0	0.00
	Never	2	7.41	0	0.00	2	22.22
Medical conditions	Cardiovascular	4	14.81	0	0.00	4	44.44
	Haematological	0	0.00	0	0.00	0	0.00
	Musculoskeletal	1	3.70	0	0.00	1	11.11
	Endocrine	0	0.00	0	0.00	0	0.00
	Respiratory	2	7.41	2	11.11	0	0.00
	Gastrointestinal	0	0.00	0	0.00	0	0.00
	Neurological		0.00	0	0.00	0	0.00
	Mental, behavioural, or neurodevelopmental disorders	6	22.22	6	33.33	0	0.00
	Cognitive	0	0.00	0	0.00	0	0.00
	Others	0	0.00	0	0.00	0	0.00
	None	9	33.33	7	38.89	2	22.22
	Not Given	4	14.81	3	16.67	1	11.11

Notes: LCA = living in a community alone, LCWO = living in a community with others, RH/AL = rehabilitation home/assisted living, UR = university residence.

**Table 5:** Hyper parameter tuning and variation ranges.

Classifier	Hyperparameter Optimised/Tuned	Range
RF	classifier__n_estimators	100, 200, 300
	classifier__max_depth	None, 10, 20, 30
	classifier__min_samples_split	2, 5, 10
	classifier__min_samples_leaf	1, 2, 4
	classifier__bootstrap	True, False
SVM	classifier__C	0.1, 1, 10, 100
	classifier__gamma	scale, auto
	classifier__kernel	rbf, linear

**Table 6:** Data set structure for machine learning implementation.

Subjects	Sessions	Trials/Session	Epochs/Trial	Epochs/Subject	Total Epochs
16	3	1	120	360	5760

**Table 7:** Analysis summary table.

Friedman Test	χ²	df	*p*-Value
EEG index 1	5.63	2	0.060
EEG index 2	3.76	2	0.153
EEG index 3	24.16	2	0.001

**Table 8:** Performance evaluation of the RF model across participants.

RF	EEG Index_1		EEG Index_2		EEG Index_3		EEG Indices_ Combined	
Subject ID	Accuracy	F1 Score	Accuracy	F1 Score	Accuracy	F1 Score	Accuracy	F1 Score
#1	0.81	0.76	0.75	0.70	0.81	0.76	0.96	0.95
#2	0.79	0.77	0.78	0.75	0.92	0.91	0.97	0.97
#3	0.83	0.80	0.76	0.70	0.78	0.73	0.92	0.90
#4	0.82	0.76	0.75	0.69	0.85	0.81	0.88	0.84
#5	0.83	0.83	0.83	0.82	0.93	0.93	0.93	0.92
#6	0.76	0.70	0.74	0.65	0.76	0.73	0.85	0.82
#7	0.86	0.82	0.82	0.76	0.68	0.57	0.86	0.83
#8	0.89	0.87	0.83	0.80	0.93	0.92	0.99	0.98
#9	0.67	0.49	0.65	0.49	0.76	0.68	0.76	0.69
#10	0.89	0.85	0.83	0.77	0.78	0.66	0.82	0.78
#11	0.76	0.73	0.72	0.67	0.78	0.76	0.86	0.83
#12	0.85	0.82	0.79	0.74	0.90	0.88	0.85	0.84
#13	0.79	0.74	0.76	0.71	0.90	0.88	0.97	0.97
#14	0.78	0.70	0.74	0.63	0.85	0.82	0.88	0.86
#15	0.83	0.8	0.81	0.76	0.79	0.76	0.92	0.90
#16	0.69	0.59	0.69	0.48	0.78	0.66	0.81	0.74
Mean	0.80	0.75	0.77	0.70	0.83	0.78	0.89	0.86
SD	0.06	0.10	0.05	0.09	0.07	0.10	0.06	0.08

SD = standard deviation.

**Table 9:** Performance evaluation of the SVM model across participants.

SVM	EEG Index_1		EEG Index_2		EEG Index_3		EEG Indices_Combined	
Subject ID	Accuracy	F1 Score	Accuracy	F1 Score	Accuracy	F1 Score	Accuracy	F1 Score
#1	0.76	0.69	0.76	0.68	0.76	0.73	0.99	0.98
#2	0.81	0.79	0.76	0.73	0.92	0.91	0.99	0.98
#3	0.82	0.79	0.79	0.74	0.76	0.69	0.93	0.92
#4	0.76	0.72	0.74	0.65	0.78	0.75	0.85	0.82
#5	0.81	0.80	0.79	0.78	0.90	0.90	0.93	0.92
#6	0.81	0.75	0.76	0.68	0.75	0.71	0.92	0.90
#7	0.82	0.78	0.81	0.77	0.68	0.60	0.90	0.89
#8	0.89	0.87	0.89	0.87	0.93	0.92	0.97	0.97
#9	0.72	0.53	0.65	0.46	0.72	0.42	0.85	0.82
#10	0.83	0.75	0.81	0.71	0.79	0.72	0.79	0.75
#11	0.74	0.70	0.75	0.72	0.75	0.72	0.92	0.91
#12	0.81	0.76	0.74	0.67	0.83	0.79	0.83	0.81
#13	0.82	0.78	0.82	0.76	0.90	0.88	0.97	0.97
#14	0.67	0.57	0.69	0.64	0.88	0.86	0.92	0.90
#15	0.89	0.87	0.81	0.76	0.82	0.79	0.90	0.89
#16	0.68	0.60	0.64	0.50	0.74	0.57	0.82	0.75
Mean	0.79	0.73	0.76	0.70	0.81	0.75	0.91	0.89
SD	0.06	0.09	0.06	0.10	0.08	0.13	0.06	0.07

SD = standard deviation.

**Table 10:** Cross-subject model classifier performance report.

	Accuracy	F1 Score
RF	79	78
SVM	81	80

## Data Availability

The data sets generated and analysed during this study are not publicly available due to the limitations imposed by the study’s ethical approval, but are available from the corresponding author on reasonable request.

## Supplementary Materials

The following supporting information can be downloaded at https://www.mdpi.com/article/10.3390/s25072072/s1.

## Abbreviations

The following abbreviations are used in this manuscript:

ML	Machine learning
GEQ	Game Engagement Questionnaire
PwD	People living with dementia
RF	Random forest
SVM	Support vector machine
UES	User Engagement Scale
EEG	Electroencephalogram
HRV	Heart rate variability
EOG	Electrooculography
ECG	Electrocardiography
GSR	Galvanic skin response
SAM	self-assessment manikin
BCI	Brain–Computer interface
AD	Alzheimer’s disease
CCT	Computerised cognitive training
POSM	partially ordered set masterI
DOL	increment/decrement one level
DNN	Deep Neural Network
ERD/ERS	Desynchronization and Event-Related Synchronisation
GAAM	Genetic Algorithm with Aggressive Mutation GAAM
