# Supplementary material for: EEG-Based Engagement Monitoring in Cognitive Games
Source: Sensors (Basel). 2025 Mar 26;25(7):2072. doi: 10.3390/s25072072 (PMC11991241; doi:10.3390/s25072072)
Supplement: Supplementary file 1 [file sensors-25-02072-s001.zip › sensors-3470059-supplementary.pdf]

Supplementary Materials

The automatic muscle noise ICA detection and rejection function in MNE has been adopted in this study. The function is only available in MNE version > 1.3. The custom code and output are detailed below.

Note: Please install the dependencies before importing them.

```
import numpy as np

import mne

from mne.preprocessing import ICA

from scipy.signal import butter, filtfilt

import matplotlib.pyplot as plt

import seaborn as sns

# Function to apply ICA and automatically remove muscle artifacts

def apply_ica(raw, label):

    # Initialize ICA using Infomax

    ica = ICA(n_components=14, method='infomax', random_state=97, max_iter=800)


    # Fit ICA to the raw EEG data

    ica.fit(raw)


    # Plot ICA components for visual inspection

    ica.plot_components(title=f'ICA Components ({label})')

    plt.show()


    # Plot band passed original EEG data before ICA

    fig_before_ica = raw.plot(n_channels=min(14, len(raw.ch_names)), scalings='auto',
show=False)
```

```
    fig_before_ica.suptitle(f'Filtered EEG Data Before ICA ({label})', size=16)
    fig_before_ica.subplots_adjust(top=0.95)
    plt.show()

    # Automatically detect muscle artifacts (requires MNE ≥ 1.3)
    try:
        muscle_inds, _ = ica.find_bads_muscle(raw)
        ica.exclude = muscle_inds  # Exclude detected muscle components
    except AttributeError:
        print("MNE version too old for automatic muscle detection. Skipping artifact removal.")
        ica.exclude = []

    print(f"Automatically excluded ICA components: {ica.exclude}")

    # Apply ICA to remove artifacts
    filtered_ica_cleaned = ica.apply(raw.copy())

    # Plot cleaned EEG data after ICA
    fig_after_ica = filtered_ica_cleaned.plot(n_channels=min(14, len(raw.ch_names)),
scalings='auto', show=False)
    fig_after_ica.suptitle(f'EEG Data After ICA ({label})', size=16)
    fig_after_ica.subplots_adjust(top=0.95)
    plt.show()

    return filtered_ica_cleaned
```

```
# Load and process EEG with ICA integration
def load_and_process_eeg(file_path, sfreq, exclude_cols, label, clip_length=120):
    eeg_data, _ = load_eeg_data_from_csv(file_path, exclude_cols)

    # Clip data to the clip length (2 minutes max)
    max_samples = clip_length * sfreq
    if eeg_data.shape[1] > max_samples:
        eeg_data = eeg_data[:, :max_samples]

    # Define channel names
    ch_names = channel_names[:eeg_data.shape[0]]

    # Create MNE Raw object
    info = mne.create_info(ch_names=ch_names, sfreq=sfreq, ch_types='eeg')
    raw = mne.io.RawArray(eeg_data, info)

    # Filter EEG data
    lowcut = 0.5
    highcut = 45.0
    filtered_data = butter_bandpass_filter(raw.get_data(), lowcut, highcut, sfreq)

    # Add standard 10-20 electrode positions
    montage = mne.channels.make_standard_montage("standard_1020")
    raw.set_montage(montage)
```

```
    # Create a new MNE Raw object for filtered data
    filtered_raw = mne.io.RawArray(filtered_data, raw.info)

    # Apply ICA and clean data
    filtered_ica_cleaned = apply_ica(filtered_raw, label)

    # Segment EEG data
    window_size = 1  # 1 second
    segments = segment_data(filtered_raw.get_data(), window_size, sfreq)

    # Calculate engagement features
    engagement_features = calculate_engagement_features(segments, sfreq)

    # Create feature column names
    feature_names = []
    for ch_name in ch_names:
        feature_names.extend([f'{ch_name}_engagement1', f'{ch_name}_engagement2',
f'{ch_name}_engagement3'])

    # Create DataFrame with features and labels
    df = pd.DataFrame(engagement_features, columns=feature_names)
    df['Label'] = label
    return df
# File-paths
easy_file_path = r'specify your file path.csv'
```

```
optimal_file_path = r'specify your file path.csv'

hard_file_path = r'specify your file path.csv'

sfreq = 128  # Set your sampling frequency here

exclude_cols = ['Timestamp', 'EEG.Counter']  # List of columns to exclude


# Process all sessions with 2min length

easy_df = load_and_process_eeg(easy_file_path, sfreq, exclude_cols, 'High',
clip_length=120)

optimal_df = load_and_process_eeg(optimal_file_path, sfreq, exclude_cols, 'High',
clip_length=120)

hard_df = load_and_process_eeg(hard_file_path, sfreq, exclude_cols, 'Low',
clip_length=120)
```

```
Creating RawArray with float64 data, n_channels=14, n_times=15360
    Range : 0 ... 15359 =      0.000 ...   119.992 secs
Ready.
Creating RawArray with float64 data, n_channels=14, n_times=15360
    Range : 0 ... 15359 =      0.000 ...   119.992 secs
Ready.
Fitting ICA to data using 14 channels (please be patient, this may take a while)
Selecting by number: 14 components
```

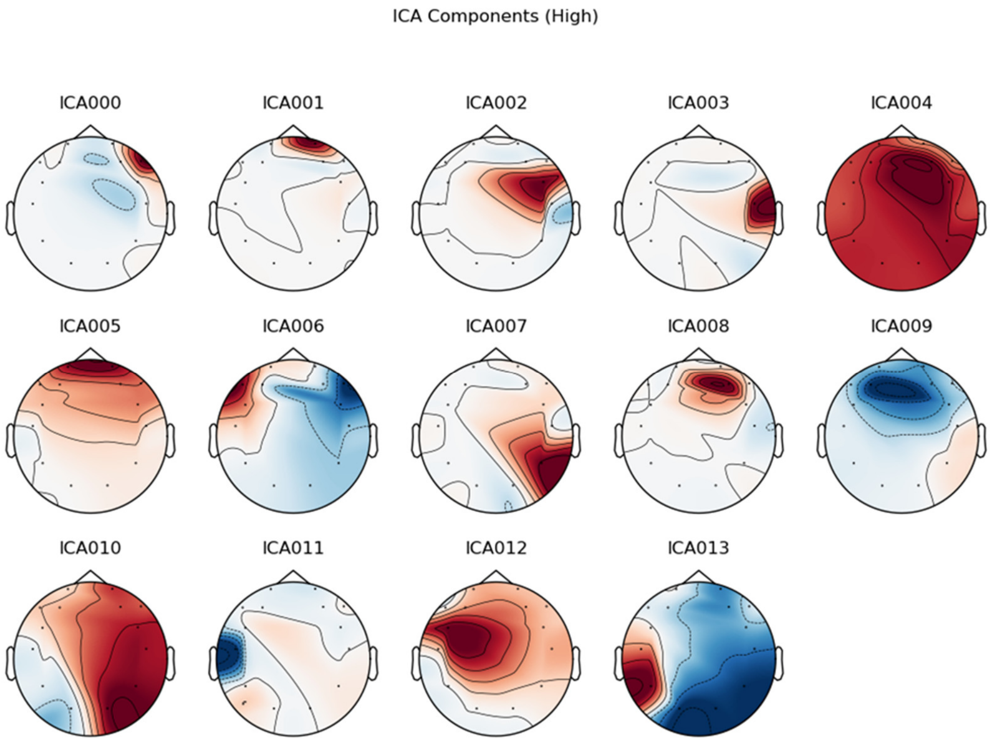

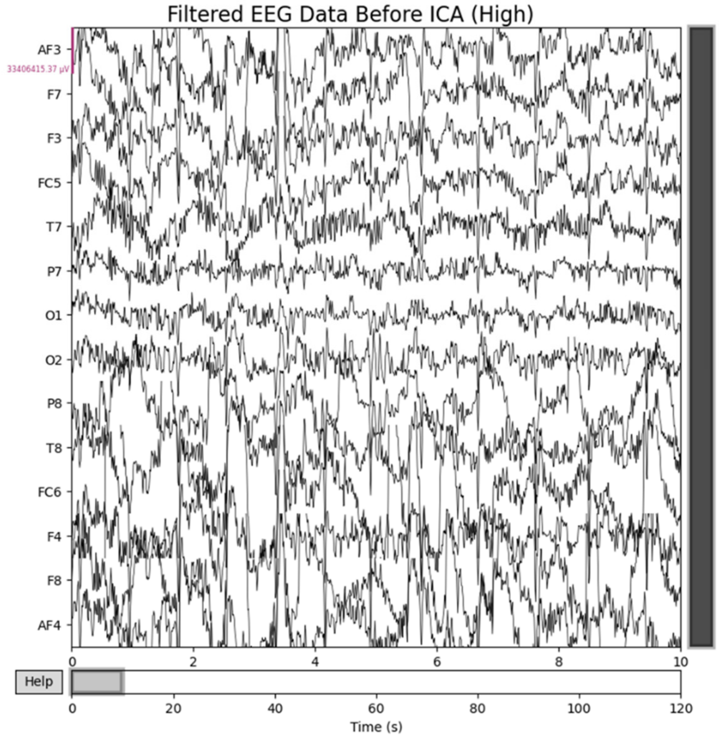

Effective window size: 16.000 (s).

Automatically excluded ICA components: [11, 12].

Applying ICA to Raw instance

Transforming to ICA space (14 components)

Zeroing out 2 ICA components

Projecting back using 14 PCA components

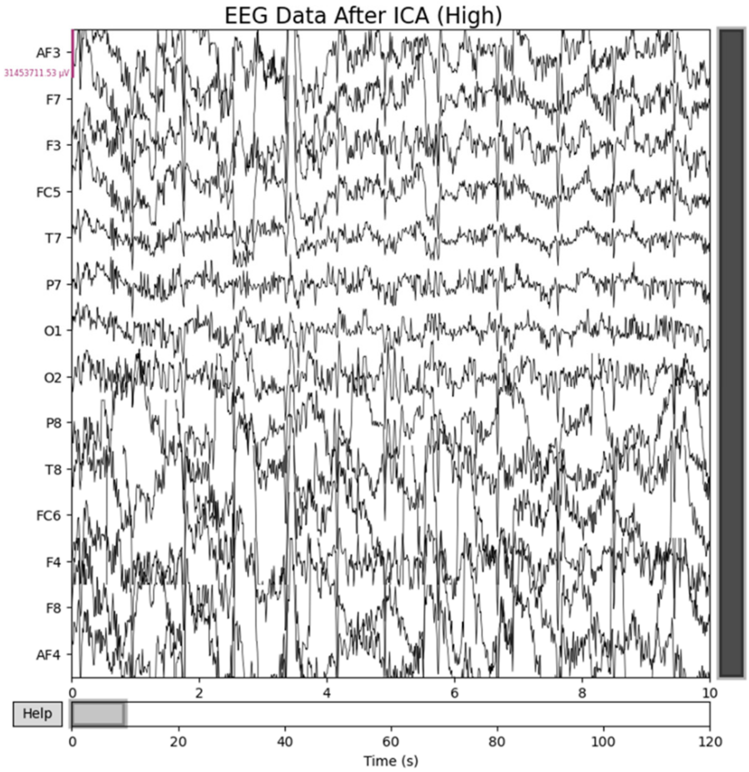

Creating RawArray with float64 data, n_channels=14, n_times=15360

Range : 0 ... 15359 = 0.000 ... 119.992 secs

Ready.

Creating RawArray with float64 data, n_channels=14, n_times=15360

Range : 0 ... 15359 = 0.000 ... 119.992 secs

Ready.

Fitting ICA to data using 14 channels (please be patient, this may take a while)

Selecting by number: 14 components

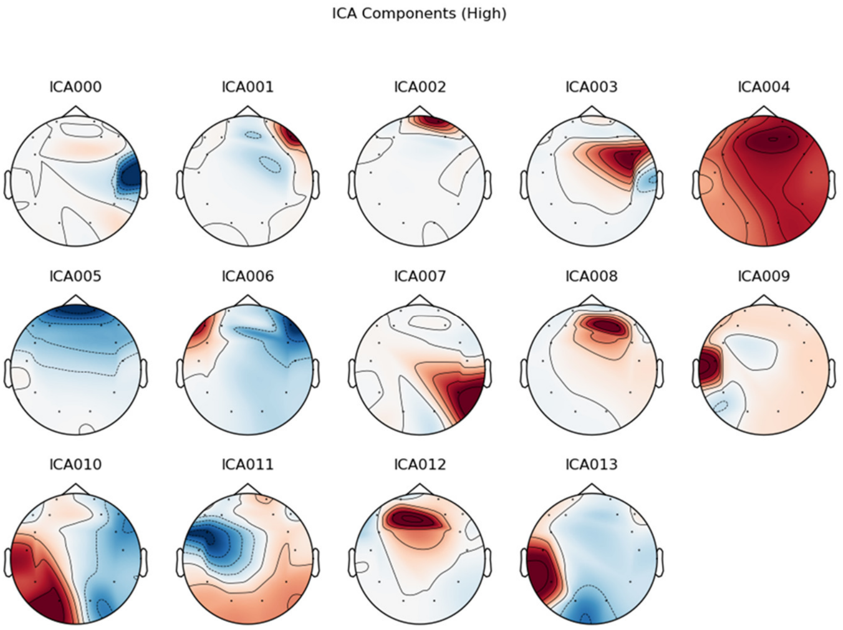

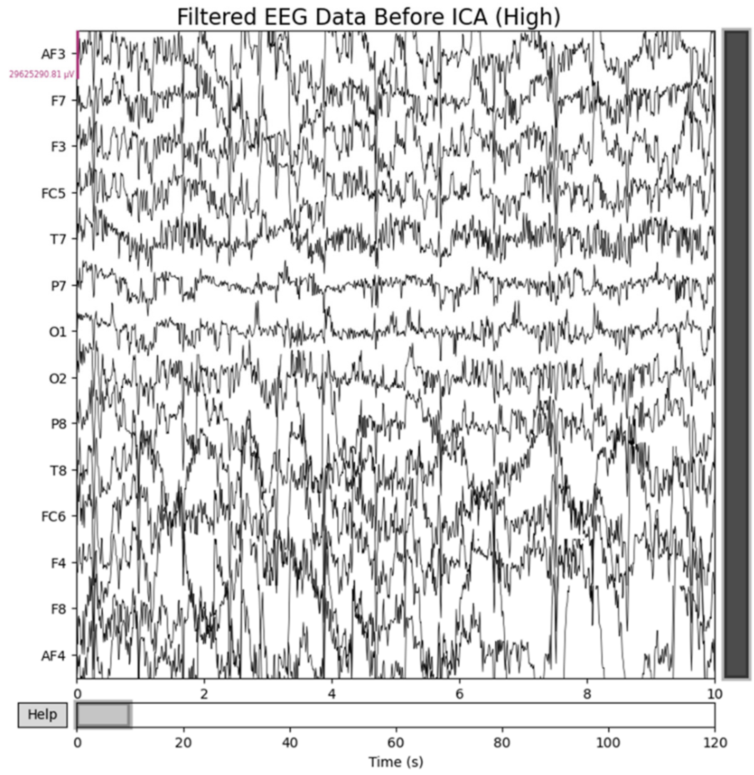

Effective window size: 16.000 (s).

Automatically excluded ICA components: [9, 13].

Applying ICA to Raw instance

Transforming to ICA space (14 components)

Zeroing out 2 ICA components

Projecting back using 14 PCA components

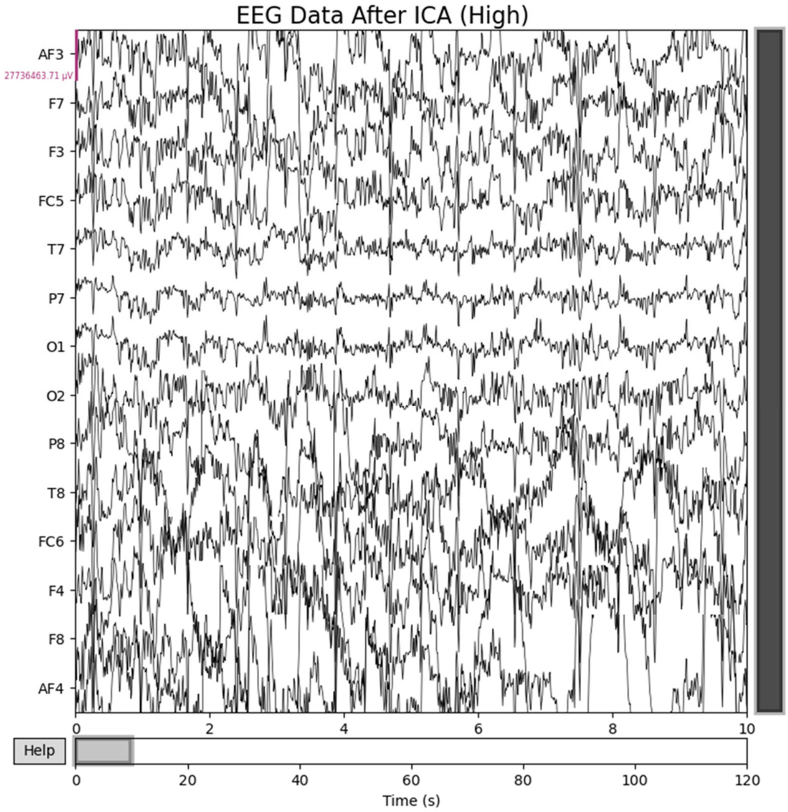

Creating RawArray with float64 data, n_channels=14, n_times=15360

Range : 0 ... 15359 = 0.000 ... 119.992 secs

Ready.

Creating RawArray with float64 data, n_channels=14, n_times=15360

Range : 0 ... 15359 = 0.000 ... 119.992 secs

Ready.

Fitting ICA to data using 14 channels (please be patient, this may take a while)

Selecting by number: 14 components

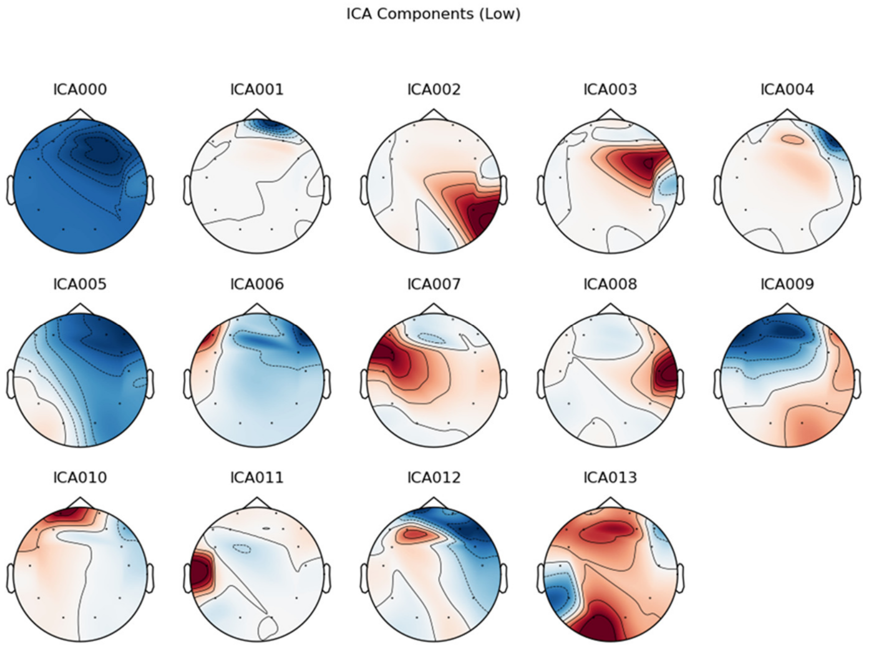

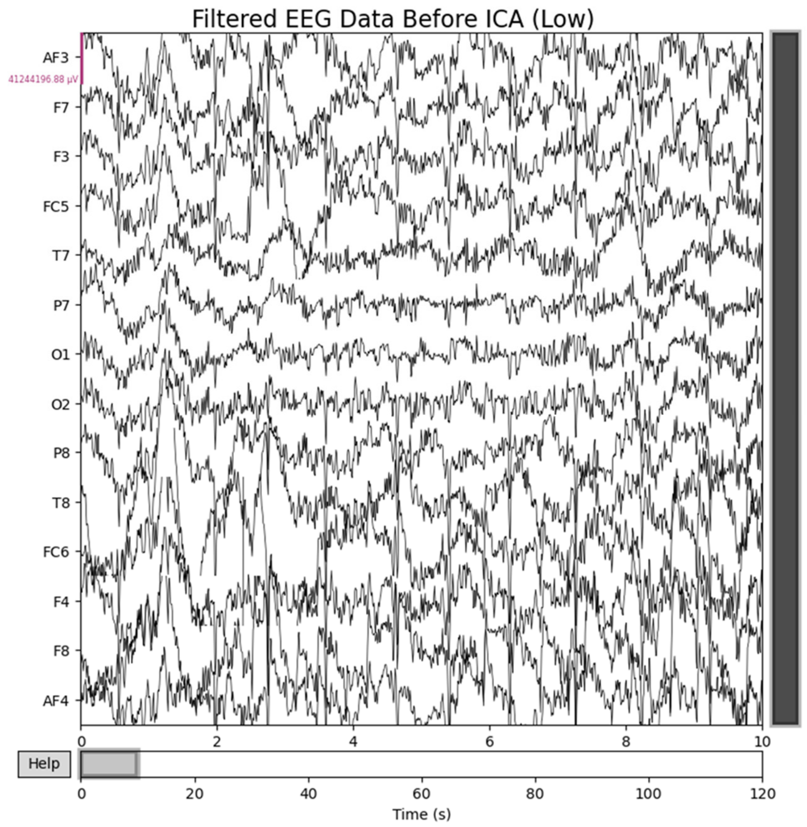

Effective window size: 16.000 (s).

Automatically excluded ICA components: [7, 10, 12, 13].

Applying ICA to Raw instance

Transforming to ICA space (14 components)

Zeroing out 4 ICA components

Projecting back using 14 PCA components

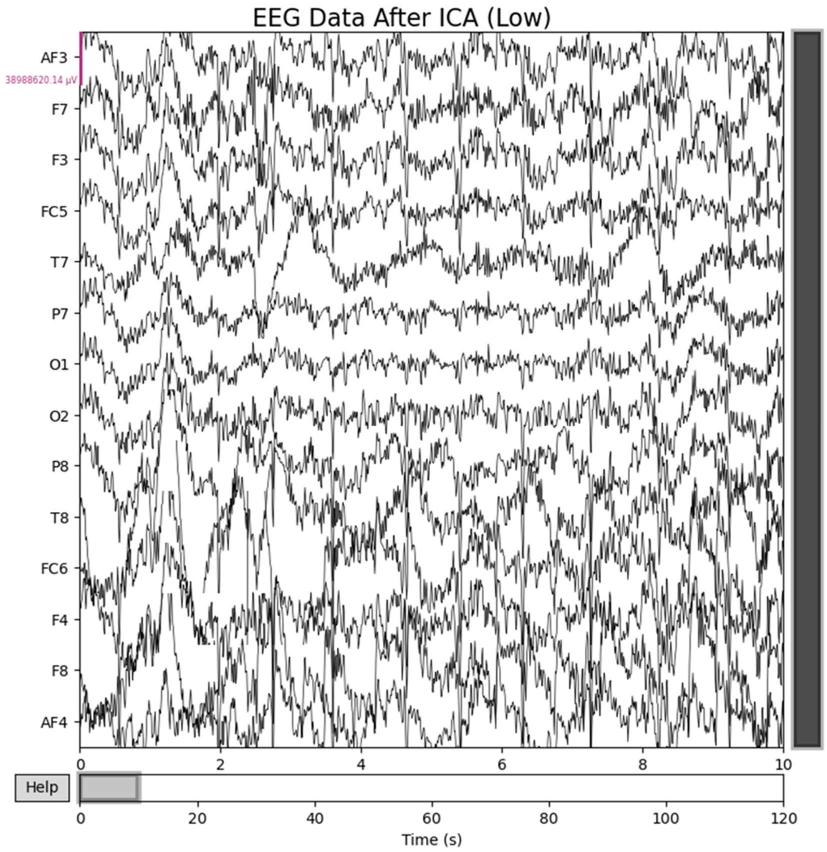

The code for implementing the signal-to-noise ratio (SNR) calculations is as follows:

```
import numpy as np
import mne
import matplotlib.pyplot as plt
import pandas as pd
from scipy.signal import butter, filtfilt
import seaborn as sns
# Column names for EEG channels
channel_names = ["AF3", "F7", "F3", "FC5", "T7", "P7", "O1", "O2", "P8", "T8", "FC6", "F4", "F8",
"AF4"]

# Load EEG data from CSV
def load_eeg_data_from_csv(file_path, exclude_cols=None):

    exclude_cols = ['Timestamp', 'EEG.Counter']  # Default list of columns to exclude
    data = pd.read_csv(file_path)
    if exclude_cols is not None:
        data = data.drop(columns=exclude_cols)
    eeg_data = data.values.T  # Transpose to get channels as rows
    return eeg_data, data.shape[0]  # Return data and the number of samples

def butter_bandpass_filter(data, lowcut, highcut, sfreq, order=4):
    """Apply a Butterworth bandpass filter to EEG data."""
    nyquist = 0.5 * sfreq
    low, high = lowcut / nyquist, highcut / nyquist
```

```
    b, a = butter(order, [low, high], btype='band')
    return filtfilt(b, a, data, axis=1)

def compute_psd_multitaper(raw, fmin=0.5, fmax=45):
    """Compute Power Spectral Density (PSD) using the multitaper method."""
    psd, freqs = mne.time_frequency.psd_array_multitaper(
        raw.get_data(),  # Extract EEG data as NumPy array
        sfreq=raw.info['sfreq'],  # Get sampling frequency
        fmin=fmin,
        fmax=fmax,
        verbose=False  # Suppress unnecessary warnings
    )
    return psd, freqs

def calculate_snr(psds, freqs, signal_bands, noise_bands):
    """Compute SNR by comparing power in signal bands vs. noise bands."""
    snr_values = []
    for psd in psds:
        signal_power = sum([np.sum(psd[(freqs >= fmin) & (freqs <= fmax)]) for fmin, fmax in
signal_bands])
        noise_power = sum([np.sum(psd[(freqs >= fmin) & (freqs <= fmax)]) for fmin, fmax in
noise_bands])
        snr = 10 * np.log10(signal_power / noise_power) if noise_power > 0 else np.nan  # Avoid
division by zero
        snr_values.append(snr)
    return np.array(snr_values)
```

```python
def segment_data(data, sfreq, max_length_sec=120):
    """Clip EEG data to a maximum of 120 seconds."""
    max_samples = int(max_length_sec * sfreq)
    return data[:, :max_samples] if data.shape[1] > max_samples else data


def plot_psd(psds, freqs, ch_names):
    """Plot Power Spectral Density (PSD) for EEG channels."""
    plt.figure(figsize=(12, 6))
    for i, psd in enumerate(psds):
        plt.semilogy(freqs, psd, label=ch_names[i])
    plt.xlabel('Frequency (Hz)')
    plt.ylabel('Power Spectral Density (dB/Hz)')
    plt.title('EEG Power Spectral Density (Multitaper)')
    plt.legend(loc='upper right', fontsize='small')
    plt.grid(True)
    plt.show()


def plot_snr(snr_values, ch_names):
    """Plot SNR values as a bar chart."""
    plt.figure(figsize=(10, 5))
    plt.bar(ch_names, snr_values, color='skyblue')
    plt.xlabel('Channels')
    plt.ylabel('SNR (dB)')
    plt.title('Signal-to-Noise Ratio (SNR) Across Channels')
    plt.xticks(rotation=45)
    plt.grid(axis='y', linestyle='--', alpha=0.7)
```

```
    plt.show()


def save_snr_to_csv(snr_values, ch_names, filename="snr_results.csv"):
    """Save SNR values to a CSV file."""
    df = pd.DataFrame({"Channel": ch_names, "SNR (dB)": snr_values})
    df.to_csv(filename, index=False)
    print(f"SNR results saved to {filename}")


def process_eeg_quality_from_csv(csv_path, sfreq, exclude_cols=None,
output_csv="snr_results.csv"):
    """Load EEG from CSV, preprocess, compute Multitaper PSD & SNR, and save results."""
    # Load data
    eeg_data, num_samples = load_eeg_data_from_csv(csv_path, exclude_cols)

    # Clip data to 120s
    clipped_data = segment_data(eeg_data, sfreq, max_length_sec=120)

    # Create MNE Raw object
    info = mne.create_info(channel_names, sfreq, ch_types="eeg")
    raw = mne.io.RawArray(clipped_data, info)

    # Apply bandpass filter (0.5 - 45 Hz)
    filtered_data = butter_bandpass_filter(raw.get_data(), 0.5, 45, sfreq)
    raw_filtered = mne.io.RawArray(filtered_data, info)

    # Compute PSD using Multitaper method
```

```
    psds, freqs = compute_psd_multitaper(raw_filtered)

    # Define signal and noise frequency bands
    signal_bands = [(4, 8), (8, 12), (13, 30)]  # Theta, Alpha, Beta
    noise_bands = [(30, 45), (50, 60)]  # Muscle artifacts, Powerline noise

    # Compute SNR
    snr_values = calculate_snr(psds, freqs, signal_bands, noise_bands)

    # Plot PSD and SNR
    plot_psd(psds, freqs, channel_names)
    plot_snr(snr_values, channel_names)

    # Save SNR results
    save_snr_to_csv(snr_values, channel_names, output_csv)

    return snr_values
# specify sampling frequency:
sfreq = 128
# Set actual file path
process_eeg_quality_from_csv(csv_path, sfreq)
csv_path = r'specify the file path'
```

Example of output:

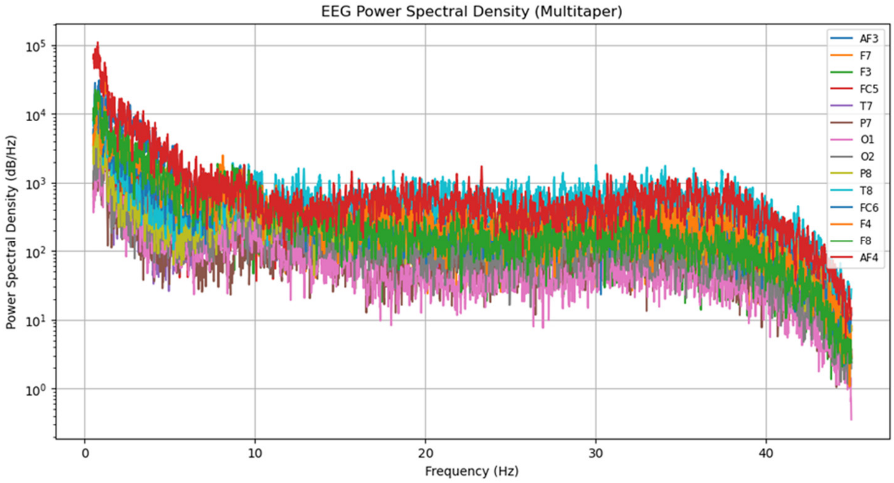

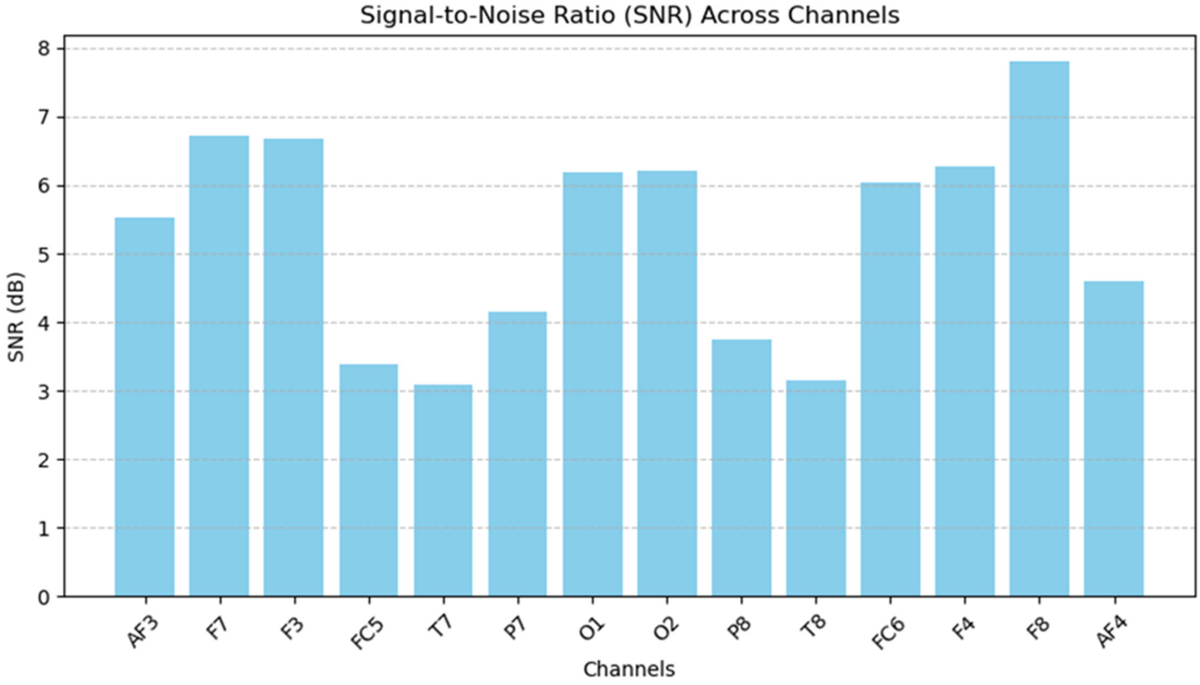

SNR results saved to snr_results.csv.

array([5.52969543, 6.70803034, 6.67661345, 3.37982364, 3.0859013 ,

4.14046169, 6.19381146, 6.19769188, 3.74609611, 3.14773111,

6.03545725, 6.26639482, 7.79205245, 4.59398601])
